# B lymphocyte responses in Parkinson’s disease and their possible significance in disease progression

**DOI:** 10.1093/braincomms/fcad060

**Published:** 2023-03-09

**Authors:** Kirsten M Scott, Yen Ting Chong, Seoyoung Park, Ruwani S Wijeyekoon, Shaista Hayat, Rebeccah J Mathews, Zachary Fitzpatrick, Pam Tyers, Georgia Wright, Jennifer Whitby, Roger A Barker, Michele T Hu, Caroline H Williams-Gray, Menna R Clatworthy

**Affiliations:** John Van Geest Centre for Brain Repair, Department of Clinical Neurosciences, University of Cambridge, Cambridge CB2 0PY, UK; Molecular Immunity Unit, Department of Medicine, University of Cambridge, Cambridge CB2 0QH, UK; John Van Geest Centre for Brain Repair, Department of Clinical Neurosciences, University of Cambridge, Cambridge CB2 0PY, UK; John Van Geest Centre for Brain Repair, Department of Clinical Neurosciences, University of Cambridge, Cambridge CB2 0PY, UK; John Van Geest Centre for Brain Repair, Department of Clinical Neurosciences, University of Cambridge, Cambridge CB2 0PY, UK; John Van Geest Centre for Brain Repair, Department of Clinical Neurosciences, University of Cambridge, Cambridge CB2 0PY, UK; Molecular Immunity Unit, Department of Medicine, University of Cambridge, Cambridge CB2 0QH, UK; Molecular Immunity Unit, Department of Medicine, University of Cambridge, Cambridge CB2 0QH, UK; John Van Geest Centre for Brain Repair, Department of Clinical Neurosciences, University of Cambridge, Cambridge CB2 0PY, UK; University of Cambridge Clinical School of Medicine, Cambridge CB2 OQQ, UK; University of Cambridge Clinical School of Medicine, Cambridge CB2 OQQ, UK; John Van Geest Centre for Brain Repair, Department of Clinical Neurosciences, University of Cambridge, Cambridge CB2 0PY, UK; Division of Neurology, Nuffield Department of Clinical Neurosciences, University of Oxford, John Radcliffe Hospital, Oxford OX3 9DU, UK; John Van Geest Centre for Brain Repair, Department of Clinical Neurosciences, University of Cambridge, Cambridge CB2 0PY, UK; Molecular Immunity Unit, Department of Medicine, University of Cambridge, Cambridge CB2 0QH, UK; Cellular Genetics, Wellcome Trust Sanger Institute, Wellcome Trust Genome Campus, Hinxton CB10 1SA, UK

**Keywords:** Parkinson’s disease, B lymphocytes, regulatory B cells, alpha-synuclein antibodies

## Abstract

Inflammation contributes to Parkinson’s disease pathogenesis. We hypothesized that B lymphocytes are involved in Parkinson’s disease progression. We measured antibodies to alpha-synuclein and tau in serum from patients with rapid eye movement sleep behaviour disorder (*n* = 79), early Parkinson’s disease (*n* = 50) and matched controls (*n* = 50). Rapid eye movement sleep behaviour disorder cases were stratified by risk of progression to Parkinson’s disease (low risk = 30, high risk = 49). We also measured B-cell activating factor of the tumour necrosis factor receptor family, C-reactive protein and total immunoglobulin G. We found elevated levels of antibodies to alpha-synuclein fibrils in rapid eye movement sleep behaviour disorder patients at high risk of Parkinson’s disease conversion (ANOVA, *P* < 0.001) and lower S129D peptide-specific antibodies in those at low risk (ANOVA, *P* < 0.001). An early humoral response to alpha-synuclein is therefore detectable prior to the development of Parkinson’s disease. Peripheral B lymphocyte phenotyping using flow cytometry in early Parkinson’s disease patients and matched controls (*n* = 41 per group) revealed reduced B cells in Parkinson’s disease, particularly in those at higher risk of developing an early dementia [*t*(3) = 2.87, *P* = 0.01]. Patients with a greater proportion of regulatory B cells had better motor scores [*F*(4,24) = 3.612, *P* = 0.019], suggesting they have a protective role in Parkinson’s disease. In contrast, B cells isolated from Parkinson’s disease patients at higher risk of dementia had greater cytokine (interleukin 6 and interleukin 10) responses following in vitro stimulation. We assessed peripheral blood lymphocytes in alpha-synuclein transgenic mouse models of Parkinson’s disease: they also had reduced B cells, suggesting this is related to alpha-synuclein pathology. In a toxin-based mouse model of Parkinson’s disease, B-cell deficiency or depletion resulted in worse pathological and behavioural outcomes, supporting the conclusion that B cells play an early protective role in dopaminergic cell loss. In conclusion, we found changes in the B-cell compartment associated with risk of disease progression in rapid eye movement sleep behaviour disorder (higher alpha-synuclein antibodies) and early Parkinson’s disease (lower levels of B lymphocytes that were more reactive to stimulation). Regulatory B cells play a protective role in a mouse model, potentially by attenuating inflammation and dopaminergic cell loss. B cells are therefore likely to be involved in the pathogenesis of Parkinson’s disease, albeit in a complex way, and thus warrant consideration as a therapeutic target.

## Teaser

Scott *et al.* report that there are changes in B lymphocytes that are associated with progression in Parkinson’s disease including increased antibodies to fibrillar alpha-synuclein in prodromal disease, decreased B cells and a protective effect of regulatory B cells. Some changes are protective; others may contribute to disease pathogenesis.

## Graphical Abstract

**Graphical Abstract:**
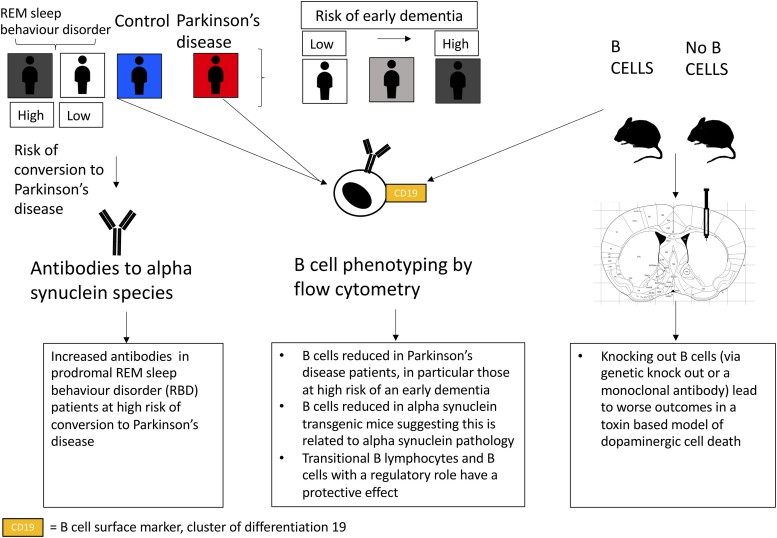

## Introduction

Parkinson’s disease is a common neurodegenerative disorder, affecting 2–3% of people over the age of 65.^1^ The disease course is complicated by dementia in nearly half of patients within 10 years of diagnosis,^2^ and there are no disease modifying treatments. Prior to developing overt Parkinson’s disease, there is also a well-described prodromal phase consisting of non-motor features such as rapid eye movement (REM) sleep behaviour disorder (RBD), constipation and hyposmia, which may precede disease onset by a decade.^3,4^ What determines whether, and at what rate, patients progress from this prodromal state to Parkinson’s disease is unknown.

The central role of alpha-synuclein pathology is well-described in Parkinson’s disease.^5-10^ There is also evidence for an immune response to alpha-synuclein with the identification of T cells specific to alpha-synuclein in Parkinson’s disease patients^6^ as well as alpha-synuclein–specific antibodies (reviewed in Scott *et al*.^7^). T-cell responses to alpha-synuclein were highest shortly after diagnosis of motor Parkinson’s disease in larger cohorts,^8^ suggesting that an adaptive immune response may peak close to disease onset. We have also shown that peripheral immune activation has prognostic significance in early Parkinson’s disease.^9^ Post-mortem studies have confirmed the presence of T-cell infiltrates in the brain in Parkinson’s disease, with greater numbers in dementia cases and their presence correlated with microglial activation and alpha-synuclein aggregates in regions relevant to cognition.^10^

B lymphocytes not only produce antibodies via terminal differentiation into plasma cells but also have antibody-independent functions. They have immunoregulatory capacity, attenuating T-cell responses via the production of IL10 and IL35.^11-14^ These so-called regulatory B cells are enriched in CD24^HI^CD38^HI^ transitional B cells^15^ and in innate CD1d^+^CD5^+^ populations.^16^ Furthermore, B cells can act as antigen presenting cells that activate CD4 T cells, produce pro-inflammatory cytokines [including granulocyte macrophage colony-stimulating factor (GM-CSF)^17^ and interleukin 6 (IL6)^18^] and respond to pathogen-associated molecular motifs (PAMPS) via Toll-like receptor or B-cell receptor engagement.^19^ B cells also comprise a quarter of meningeal immune cells in homoeostasis,^20^ and more recently, IgA-producing plasma cells (typically found at mucosal surfaces) have been identified in the meningeal dural sinuses, with evidence that they are ‘trained’ in the gut and then home to the meninges to protect the brain.^21^ Given the pleiotropic roles of B cells across the immune response, including CD4 T-cell activation and their presence in the meninges, we reasoned that they may be involved in the pathogenesis and progression of Parkinson’s disease.

It is relatively well-established that B lymphocytes are reduced in Parkinson’s disease,^22-25^ although this is not replicated in all studies.^26^ More recent studies have shown that alterations in B-cell subsets are also relevant with an increase in effector activity (increased cytokine production or memory B cells) in patients compared to controls^24,26^ or a decrease in regulatory B cells.^27^ Single-cell RNA sequencing of peripheral blood in a small sample (eight patients, six controls) showed clonal expansion of memory B cells with upregulation of major histocompatibility complex (MHC) Class II highlighting relevance of B cells as antigen presenting cells and their potential role in activating T cells.^28^ It is unclear what role these changes have in disease progression.

We therefore sought to examine antibody responses to alpha-synuclein species in a prodromal RBD and early Parkinson’s disease patient cohort together with well-matched controls, along with comprehensive phenotyping of peripheral B cells. We performed supportive mechanistic studies in relevant mouse models of Parkinson’s disease.

## Materials and methods

The clinical studies were approved by the Cambridgeshire Research Ethics Committee (03/303) and South Central-Oxfordshire A Research Ethics Committee (ID 188167). Written informed consent was obtained from participants in accordance with the Declaration of Helsinki.

### Study participants

Patients with idiopathic RBD were recruited through the Oxford Parkinson’s Disease Centre (OPDC) Discovery Study from sleep disorder clinics at the John Radcliffe Hospital in Oxford, Papworth Hospital in Cambridge and Sheffield Teaching Hospital. RBD diagnosis, as described by Barber *et al*.,^29^ was based on polysomnographic evidence according to the International Classification of Sleep Disorders criteria.^30^ Idiopathic RBD patients were gender matched to Parkinson’s disease patients and controls and stratified into low and high conversion risk prodromal Parkinson’s disease groups (see Table 1). A probability score was calculated for prodromal Parkinson’s disease for each REM sleep behaviour participant at their baseline assessment, based on the Movement Disorder Society (MDS) criteria.^31^ A threshold of 80% was applied for stratification: patients with scores >80% were stratified into the ‘high-risk’ prodromal Parkinson’s disease group, while those with scores lower than 80% were stratified into the ‘low-risk’ prodromal Parkinson’s disease group. Cases who were current users of immunomodulatory therapies or with known autoimmune disorders were excluded.

**Table 1:** Participant clinical and demographic information (antibody study)

Variable	Control (*n* = 50)	Low-risk RBD (*n* = 30)	High-risk RBD (*n* = 49)	Parkinson’s disease (*n* = 50)
Age	67.72 (5.09)	62.10 (9.52)^a^	67.72 (5.93)	68.01 (6.47)
Gender (% male)	55	80	87.8	66
Disease duration				3.2 (2.6)
MDS-UPDRS				34.3 (13.65)
MMSE				28.52 (2)
LEDD (mg)				517.37 (367.66)

MDS-UPDRS, Movement Disorders Society Unified Parkinson’s Disease Rating Scale; MMSE, Mini-Mental State Examination; LEDD, levodopa equivalent daily dose; RBD, REM sleep behaviour disorder.

^a^Age was compared using a one-way ANOVA with *post hoc t*-tests. Low-risk RBD patients were significantly younger than the other groups (*P* < 0.002). The gender distribution was compared with chi-square tests. There was no significant difference between groups.

Parkinson’s disease cases were recruited from the Cambridge Parkinson’s Disease Research Clinic. Inclusion criteria were fulfilment of the UK Parkinson’s disease Brain Bank Criteria for a diagnosis of Parkinson’s disease, age 55–80 years and early-stage disease (Hoehn and Yahr stage ≤ 2). Exclusion criteria included the presence of other neurodegenerative disorders, chronic inflammatory/autoimmune disorders, current infection, surgery in the last month, vaccinations in the previous 3 weeks or recent use of anti-inflammatory/immunomodulating medications (steroids in the previous 3 months, high-dose aspirin >75 mg in the previous 2 weeks, ibuprofen and other non-steroidal anti-inflammatory drugs in the previous 2 weeks or any long-term immunosuppressants). Parkinson’s disease cases were stratified into groups at high, intermediate and low risk of developing an early dementia using microtubule-associated protein tau (MAPT) genotype and neuropsychological predictors identified previously in a longitudinal cohort study of incident Parkinson’s disease.^32^ See Supplementary Methods for further details.

Immunophenotyping controls were recruited from the National Institutes of Health Research (NIHR) Cambridge BioResource (www.cambridgebioresource.group.cam.ac.uk) and were age, gender and MAPT genotype matched to Parkinson’s disease patients. Further controls for the antibody study were recruited opportunistically from spouses of patients. Controls had no history of neurological disease, memory problems or depression. Exclusion criteria were the same as for the patients. See Supplementary Materials and Methods for details regarding blood sampling.

### Antibody assays

Antibody levels to relevant antigens of interest were measured using custom Mesoscale Discovery (MSD) electrochemiluminescence assays in RBD, early Parkinson’s disease patients and controls. Antigen preparation is described in full in the Supplementary Methods. All serum samples were run in duplicate. Streptavidin plates (MSD) were blocked with 150 μL of 3% bovine serum albumin (BSA) (Millipore, cat. no. 82–045-1) in phosphate-buffered saline (PBS) for 1 h on a shaker at 4000 revolutions per minute (RPM). They were washed three times with 150 μL 0.05% Tween 20 (Sigma-Aldrich P1279) PBS. The plates were then coated with 25 μL of antigen and incubated overnight at 4°C, then washed three times with 0.05% Tween 20 PBS. Calibrator antibodies were prepared as described in the Supplementary Methods. Serum was diluted 1:25 in 1% BSA in PBS. Diluted serum or calibrator was added in volumes of 25 μL. Plates were incubated for 1 h at room temperature on a shaker at 4000 RPM. They were washed again with 0.05% Tween 20 PBS three times. MSD sulfo-tag anti-human antibody was then added to the test wells and MSD sulfo-tag anti-mouse or anti-rabbit to the calibrator wells at a concentration of 1:500. The plates were then incubated at room temperature for an hour on a shaker at 4000 RPM. After incubation, plates were washed three times with 0.05% Tween 20 PBS and 150 μL of 1× read buffer was added. Electrochemiluminescence was read on an MSD plate reader (Sector S 600). Absolute concentrations were calculated from standard curves.

### Serum IgG/CRP and BAFF measurements

The MSD human total IgG panel (K15203D) was used to measure total IgG, and the MSD V-

PLEX human C-reactive protein (CRP) (K151STD) for CRP. B-cell activating factor of the tumour necrosis factor receptor family (BAFF) was measured using the human BAFF ELISA Kit (ab119579, Abcam) as per the manufacturer’s instructions. Samples were run in duplicate.

### PBMC isolation and immunophenotyping in Parkinson’s disease patients and controls

Blood samples for peripheral blood mononuclear cell (PBMC) isolation from matched Parkinson’s disease and control pairs were collected and processed on the same day and the same time (see Supplementary Materials and Methods for further details). PBMCs were isolated from heparinized blood using a Ficoll gradient within 2 h of phlebotomy. Isolated PBMCs were left to block in buffer for 10 min at 4°C (0.1% BSA, Probumin. Millipore, cat. no. 82-045-1; 0.01% sodium azide, Sigma-Aldrich, cat. no. S2002 made up in PBS; 2% mouse serum, Sigma-Aldrich, cat. no. M5905). Next, 0.5 μL of zombie aqua (Biolegend 423101) was added and incubated for 15 min prior to addition of surface staining antibodies (see Supplementary Methods). Samples were incubated at 4°C for 30 min. The PBMCs were then washed twice before being fixed in 2% paraformaldehyde (PFA) for 20 min. Flow cytometry was run within 2–4 h. Flow Cytometry Standard (FCS) output files were imported into FlowJo software (version 10.5.0), which was used for gating of the cell populations, which was done blinded to group. All flow cytometry was run on the BD Fortessa II flow cytometer with a minimum of 5000 B cells recorded.

### B lymphocyte stimulation

PBMCs were defrosted (see Supplementary Methods) and cultured for 48 h in 200 μL of RPMI cell culture media with glutamine (Gibco 21875-034) in a 96-well round bottom plate. The test wells were stimulated with either CpG DNA (1:10 000) (Cambridge Bioscience #Hycult HC4039) and CD40 ligand (1:5000) (R and D systems, 6245-CL-050) or alpha-synuclein fibrils for 48 h. Five hours prior to removal from culture, phorbol 12-myristate 13-acetate (PMA) (50 ng/mL) (Sigma-Aldrich #P8139), ionomycin (500 ng/mL) (Sigma-Aldrich #I0634) and brefeldin A (BFA) (5 μg/mL) (BioLegend #420601) (PIB) were added to the CD40L/CpG wells and to separate intermediate (PIB) wells. BFA was added to the alpha-synuclein and unstimulated wells.

Cells were removed from culture at 48 h and centrifuged at 350*g* for 5 min. Surface staining was done as per the protocol above excluding the fixation step. Cells were fixed and permeabilized prior to intracellular staining using the eBiosciences fixation and permeabilization kit as per manufacturer’s instructions [Fix/Perm concentrate (00-5123-43) Fix/Perm Diluent (00-5223-56) and 10 × Perm buffer (00-8333-56)]. See Supplementary Methods for a list of antibodies. Flow cytometry was run within 2–4 h.

### Mouse experiments

#### Ethical statement

All animal studies were done in accordance with the Animal (Scientific Procedures) Act 1986 (UK).

C57bl/6 mice were obtained from the Jackson Laboratories or from in house breeding. B-cell–deficient μMT mice were obtained in house. They have a mutation affecting the immunoglobulin heavy chain (μ chain) that means that they don’t express membrane bound IgM and are unable to produce B cells beyond the pre-B stage. Tissue from MI-2 mice was given to us by Michal Wegrzynowicz and Maria Grazia Spillantini (University of Cambridge). The MI-2 mouse is a model of Parkinson’s disease created by expressing a truncated version of human alpha-synuclein (asyn 1–120) on the tyrosine hydroxylase (TH) promotor^33^ on the background of a C57bl/6J mouse with a spontaneous deletion in SNCA (C57BL/6J OlaHsd mice (Snca^−/−^). These mice were bred in house; SNCA knockout mice and C57bl/6 mice were used as littermate controls.

Mice overexpressing alpha-synuclein on the Thy1 promotor (mThy1-hSNCA, Line 15 mice) were obtained from the Jackson Laboratories (https://www.jax.org/strain/017682). Mice for experiments were bred from a hemizygous carrier and a non-carrier. All mice were genotyped using JAX protocols (available at https://www.jax.org/strain/017682). Wild-type controls and transgenic littermates were co-housed where possible.

#### Mouse PBMC staining

See Supplementary Materials for blood sampling and lysis protocols. Cells were washed twice with 1 mL of PBS then spun for 10 min at 2000 RPM between washes. Rat serum was added for blocking (Aviva Systems Biology, RA01679). Conjugated cell surface antibodies and a live/dead stain were then added to the cell suspension and incubated for an hour at a concentration of 1:200. See Supplementary Methods for antibodies. The cells were washed in 5 mL of PBS, re-suspended in FACS fix (1% PFA) and stored at 4°C. They were run on the flow cytometer (BD LSR Fortessa) within 24 h.

#### 6-OHDA model

Mice were anaesthetized to a surgical depth using isoflurane (2 L/min) (Baxter, Newbury, UK). Their head was shaved, and the underlying skin was cleaned to create a sterile field. They were placed in a stereotaxic frame. The needle was positioned to target the striatum [anteroposterior (AP) 0.5 mm, mediolateral (ML) 2 mm, ventral (V) 3.0 mm from bregma] where 1 μL of 6-hydroxydopamine (6-OHDA) (5 μg/μL) (Sigma-Aldrich, cat no. 162957) was injected at a rate of 0.5 μL/min.

#### B-cell depletion

B-cell depletion was performed using an anti-CD20 monoclonal antibody (mIgG2a, kind gift from Biogen, 18B12) at a dose of 10 mg/kg or control antibody at the same concentration [anti-human CD20, IgG1 Truxima/Rituxan (Rituximab)] via the tail vein. Effective depletion lasts for 5–8 weeks at this dose.^34^ The human anti-CD20 was chosen as a control given it is an IgG antibody but has no antigen-specific biological effect in the mouse and both antibodies have mouse variable regions (with rituximab having human a constant region).

#### Behavioural testing

The rotarod was used as a measure of motor performance. This was run at weekly intervals both pre-surgery and post-surgery for the experiments using the toxin-based models (see Supplementary Methods).

#### Brain sectioning and immunohistochemistry

See Supplementary Materials and Methods section for mouse culling, tissue fixation and immunohistochemistry protocols.

#### Stereological estimation of TH^+^ neuron density

The substantia nigra was defined anatomically as described in previous work in the Barker Lab.^35^ See Supplementary Methods for full details of the stereological estimation of TH^+^ cell counts.

### Statistical analysis

#### Antibody study

For the antibody study, standard curves were generated in MSD Discovery Workbench, and the calculated titres were exported into Excel (version 16.16.2). Samples with an unacceptably high coefficient of variation (CV) were removed from the analysis (CV > 20%) (see Supplementary Table 1). Sample CV was calculated using the following formula:


$$\mathrm{Sample}\;\mathrm{CV}=\frac{\sigma }{\mu }$$

where σ is the standard deviation of the two duplicates and μ is the mean.

A mean CV of all remaining samples was calculated for each assay to provide a measure of overall intra-assay variability. Each plate included a control serum sample, which was the same across all plates and assays. Inter-assay (between-plate) variability for each assay was assessed by computing the CV of the concentrations of control serum across all plates of the respective assay (Supplementary Table 2). Antibody titres were normalized to the concentration of control serum on the respective plate to overcome inter-assay variability and to ensure the reliability of results. After exclusion of samples with a high CV, the mean intra-assay CV for the BAFF assay was 10.3%. All other assays had a mean CV of less than 10%.

Percentage recovery was calculated using the standards of known concentration. Mean age differences between groups were examined using a one-way analysis of variance (ANOVA), followed by the Bonferroni *post hoc* multiple comparisons test, while gender proportion differences were assessed using the chi-square test. Relationships between variables were explored using Pearson correlation coefficients. Between-group differences for all assays were assessed for statistical significance using a one-way analysis of covariance (ANCOVA) with age and gender included as covariates followed by a Bonferroni *post hoc* multiple comparison test. Independent samples *t*-tests were performed to compare means of the control group against the Parkinson’s disease group and low-risk versus high-risk prodromal group. An adjusted value of *P* < 0.05 was considered statistically significant for all analyses.

#### B-cell phenotyping and stimulation studies

Flow cytometry data were analysed using FlowJo (version 7 for Mac OS). Subsequent data analysis was performed using IBM SPSS version 25. The study was primarily designed to facilitate paired comparisons between the risk groups (high, intermediate and low) and their matched controls, with paired samples processed on the same day. Patient and control demographic variables were compared using paired *t*-tests for continuous parametric variables (or non-parametric equivalents where appropriate) and chi-square tests for categorical variables. The Kolmogorov–Smirnov test was used to compare data to a normal distribution. Clinical variables were compared across the three defined risk groups (high risk, intermediate and low risk) using a one-way ANOVA. *Post hoc t*-tests were carried out if appropriate. Figures show the mean and SD unless stated otherwise. Heat maps were generated using the gplot function in R. FCS output files from flow cytometry were imported into FlowJo software (version 10.5.0) that was used for initial gating of the cell populations, which was done blinded to group. Comparisons between groups were done between matched pairs using paired *t*-tests. Pearson correlation coefficients were used as above for relationships between B-cell measures and clinical parameters. In order to look at relationships across variables in a more robust manner, a regression model was constructed using all of the B lymphocyte subsets that were negatively associated with motor Unified Parkinson’s Disease Rating Scale (UPDRS) scores given that they were correlated with each other (using SPSS). All of the variables were entered into the model at the same time.

For the B-cell stimulation experiments, between-group comparisons were done either using paired *t*-tests (or non-parametric tests where appropriate) for patient versus control comparisons or unpaired *t*-tests between risk groups. Mixed measures ANOVAs were used to explore variation in each stimulation condition with IL10, IL6 and CD25 as the repeated measures (three levels: unstimulated, stimulated and alpha-synuclein) and risk group as the independent variable. Greenhouse–Geisser correction was applied to the degrees of freedom where the assumption of sphericity was violated. A correlation matrix was also constructed to explore relationships between measured variables and outcome measures (as in the previous study). Multiple linear regression models were run using SPSS.

#### Animal models

Behavioural experiments were analysed using two-way ANOVAs with motor performance as the dependent variable and genotype as an independent variable. Between-group differences in B lymphocyte proportions or subsets in the animal models were tested using either unpaired *t*-tests (for parametric data), Mann–Whitney U tests or ANOVA depending on the number of comparisons.

## Results

### Increased alpha-synuclein fibril antibodies in RBD patients at high risk of Parkinson’s disease conversion

**Figure 1:**
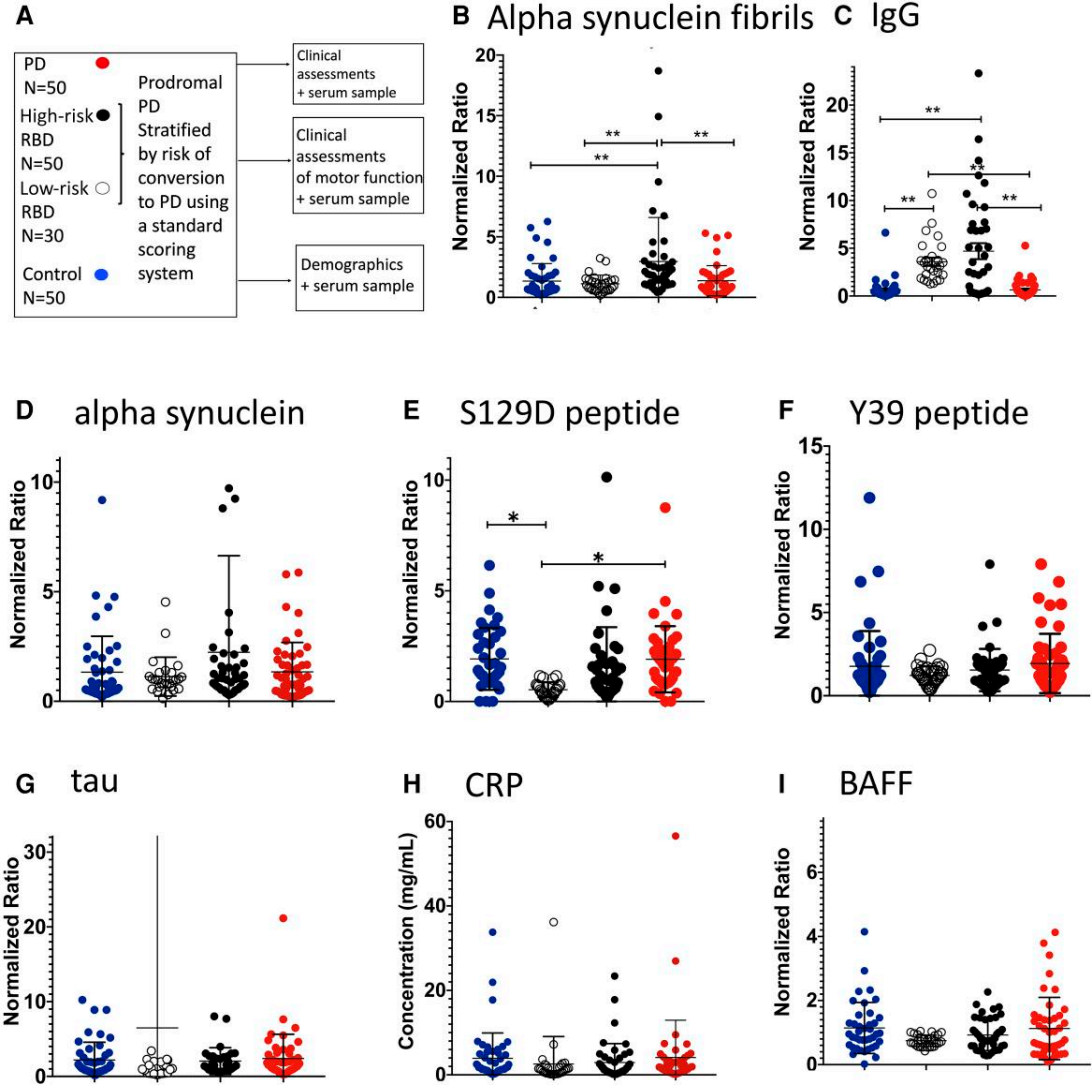
**Antibodies to disease relevant proteins and serum markers in a stratified cohort of patients with RBD, early Parkinson’s disease and controls.** (**A**) Serum and clinical assessments obtained from a stratified RBD cohort (high risk of conversion to Parkinson’s disease versus low risk) as well as early Parkinson’s disease and healthy controls. Risk of conversion to Parkinson’s disease was stratified using a standard scoring system including environmental risk (e.g. pesticide exposure), age and clinical parameters (motor and non-motor symptoms).^31^ Figures displayed as mean (standard deviation). (**B**) Antibodies to alpha-synuclein fibrils [ANCOVA *F*(5,162) = 4.275, *P* < 0.001 with *post hoc t*-tests with Bonferroni correction showing: high risk versus control *P* = 0.002, high risk versus low risk *P* = 0.002, high risk versus Parkinson’s disease, *P* = 0.003]. (**C**) IgG overall [ANCOVA, *F*(5,149] = 11.41, *P* < 0.001 with *post hoc* Bonferroni testing showing *P* = 0.001 for control versus low-risk and high-risk and for Parkinson’s disease versus low risk and high risk]. (**D**) Antibodies to alpha-synuclein. (**E**) antibodies to S129D peptide [ANCOVA *F*(5,139) = 3.14, *P* = 0.01, *post hoc* Bonferroni (low risk versus control, *P* = 0.005; low risk versus Parkinson’s disease, *P* = 0.004; low risk versus high risk, *P* = 0.05 (ns)]. (**F**) Antibodies to Y39 peptide of α synuclein. (**G**) Antibodies to tau. (**H**) CRP levels across the groups. (**I**) BAFF levels across the groups. RBD, REM sleep behaviour disorder; PD, Parkinson’s disease; BAFF, B-cell-activating factor; CRP, C reactive protein. One-way ANCOVA was performed to assess between-group differences in means. Differences displayed are those shown on *post hoc* testing. *P < 0.05 and **P < 0.001 indicate statistical significance compared to Parkinson’s disease and control groups after Bonferroni adjustment.

Serum samples from a REM sleep behaviour cohort stratified by risk of progression to overt Parkinson’s disease (high risk versus low risk) and from early-stage Parkinson’s disease patients and controls (Table 1 and Fig. 1A) were applied to a custom MSD assay for antibodies to disease relevant proteins, including alpha-synuclein, alpha-synuclein fibrils, S129D peptide, Y39 peptide and tau. Total serum IgG, CRP and BAFF were also measured (Fig. 1B–I). The RBD low conversion risk group was significantly younger than the other groups (mean age = 62.10, SD 9.52 versus 67.72, 67.72 and 68.01 in the control, RBD high-risk and Parkinson’s disease groups, respectively; see Table 1). Subsequent analyses were age-adjusted. We found increased levels of antibodies to alpha-synuclein fibrils in RBD patients at high risk of conversion to Parkinson’s disease, compared with those at low risk (Fig. 1B).

This suggests a humoral response to an aggregated form of the protein is present prior to the development of overt Parkinson’s disease. There was also an overall increase in normalized IgG titres in both low- and high-risk prodromal groups (Fig. 1C). In the RBD low-risk group, there were lower levels of antibodies to S129D peptide compared with other groups (Fig. 1E). There were no differences in levels of antibodies to other proteins/peptides and no difference in serum BAFF or CRP levels between groups (Fig. 1).

Within the RBD cohort, the probability of Parkinson’s disease conversion (MDS score) showed a significant positive correlation with normalized titres of alpha-synuclein fibril antibodies (*r* = 0.25, *P* = 0.035, Supplementary Fig. 1A). However, in early Parkinson’s disease patients, neither protein-relevant antibodies nor IgG and BAFF showed correlations with clinical measures (Supplementary Fig. 1B).

### Parkinson’s disease patients have reduced B cells, particularly in a subset enriched for regulatory B cells, and regulatory B cells are negatively correlated with disease severity

**Figure 2:**
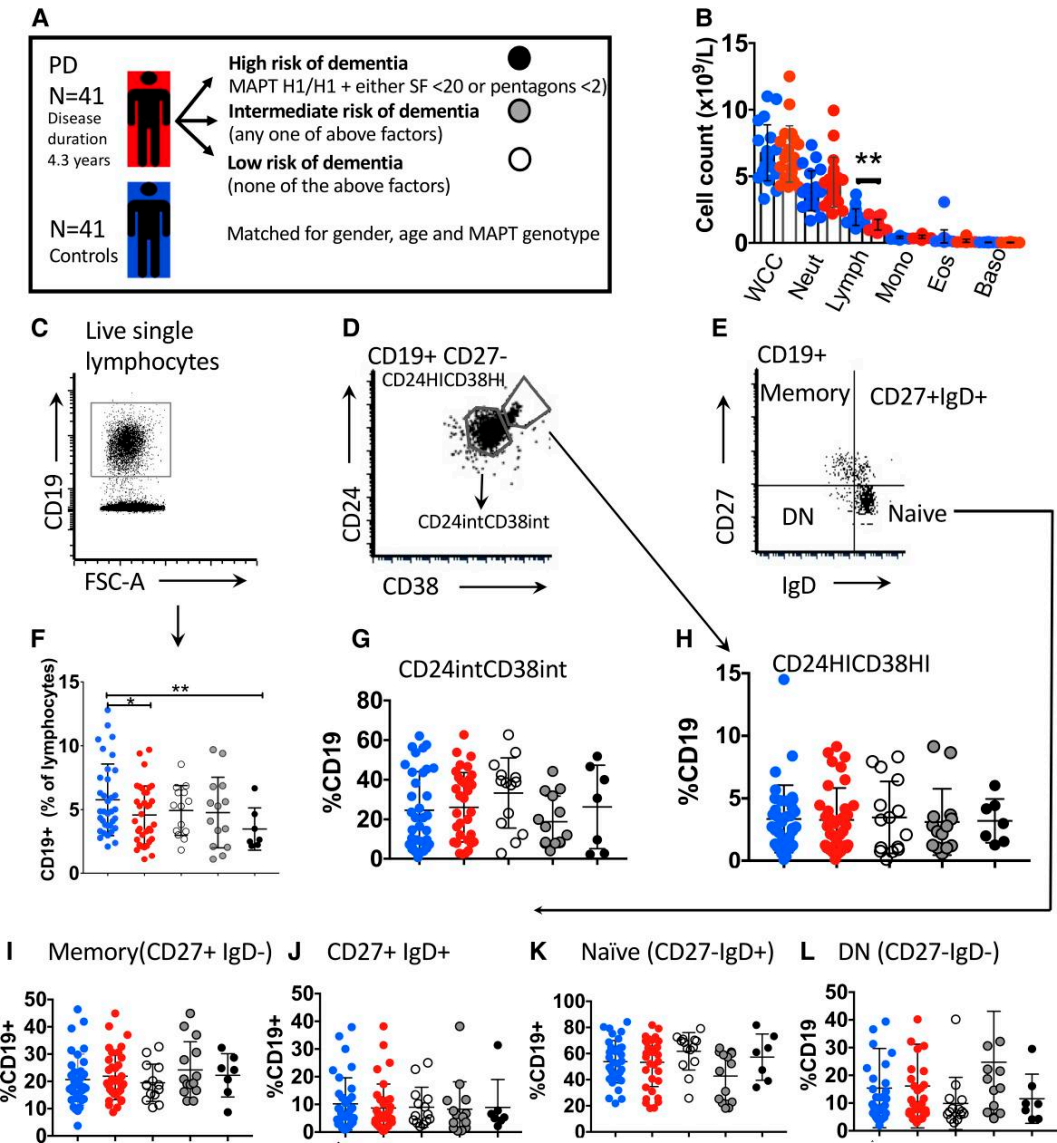
**B lymphocyte phenotyping in a Parkinson’s disease cohort stratified by risk of early dementia with age, gender and MAPT genotype-matched controls.** (**A**) Parkinson’s disease patients were stratified by their risk of an early dementia and matched to controls. MAPT, microtubule-associated protein tau (H1 haplotype is associated with increased dementia risk in Parkinson’s disease^2^); SF, semantic fluency; pentagons, pentagon copying test; PD, Parkinson’s disease. Samples from each patient–control pair were run at the same time to minimize inter-assay variability. (**B**) Full blood count data showing that patients had significantly lower lymphocyte counts than matched controls (graph shows mean and SD). (**C–L**) Representative gating of major B lymphocyte subsets with group comparisons (see main text for subsets). (**C**) Gating showing CD19^+^ B lymphocytes, and in **F**, the percentage of B lymphocytes is reduced overall in patients compared to controls [paired *t*-test *t*(34) = 2.08, *P* = 0.04]. Paired *t*-tests between Parkinson’s disease patients and matched controls showed that there was a significant difference in the percentage of B cells between high-risk patients and their matched controls but not between the other groups and their matched controls [*t*(6) = 3.42, *P* = 0.01]. (**D**) Gating showing CD27^−^CD24^HI^CD38^HI^ IL10-producing subpopulation and an intermediate population. The differences between groups are shown in **G** and **H** (comparisons not statistically significant). (**E**) Gaiting using CD27 and IgD showing memory B cells (CD27^+^IgD^−^), Naïve (CD27^−^IgD^+^), double negative (DN) and CD27^+^IgD^+^ subpopulations. Differences between groups are shown in I (Memory B cells), J (CD27^+^IgD^+^), K (Naïve) and L double negative. These were not statistically significant.

Peripheral B cells were immunophenotyped in 41 early Parkinson’s disease patients and 41 age-, gender- and MAPT genotype-matched controls (Fig. 2A and Table 2). Serum from some of these patients was also included in the antibody study (although not in sufficient numbers to allow us to stratify the antibody titres by dementia risk). Total lymphocyte numbers were reduced in Parkinson’s disease patients compared with controls (Fig. 2B), with similar numbers of T cells, but significantly lower B-cell numbers in Parkinson’s disease patients, particularly in those at high risk of developing an early dementia (Fig. 2C and F).

**Table 2:** Participant demographic and clinical information (B cell phenotyping)

Variable	Controls (*n* = 41)	Parkinson’s disease (*n* = 41)				*P*-value
		All	High risk (*n* = 9)	Intermediate risk (*n* = 14)	Low risk (*n* = 18)	
Age	68.1 (5.6)	68.4 (6.3)	69.3 (4.8)	70.4 (6.7)	66.3 (6.3)	0.74*
Gender (% male)	68.3	68.3	66.7	78.6	61.1	1*
Disease duration	N/A	4.24 (1.2)	4.3 (1.1)	4.3 (1.1)	4.23 (1.3)	0.995
MDS-UPDRS motor score	N/A	35.23 (12.3)	42.9 (12.0)	35.43 (12.7)	31 (10.8)	0.06
ACE-R	N/A	93.0 (8.3)	82 (11.03)	95.1 (4.5)	96.8 (2.3)	0.000001
Levodopa equivalent daily dose (mg)	N/A	591.5 (292.9)	389.2 (268.7)	560.3 (257.8)	717.0 (279.3)	0.017

MDS-UPDRS, Movement Disorders Society Unified Parkinson’s Disease Rating Scale; ACE-R, Addenbrooke’s Cognitive Examination Revised. *Participants were age and gender matched, asterisk indicates non-significant values.

**Figure 3:**
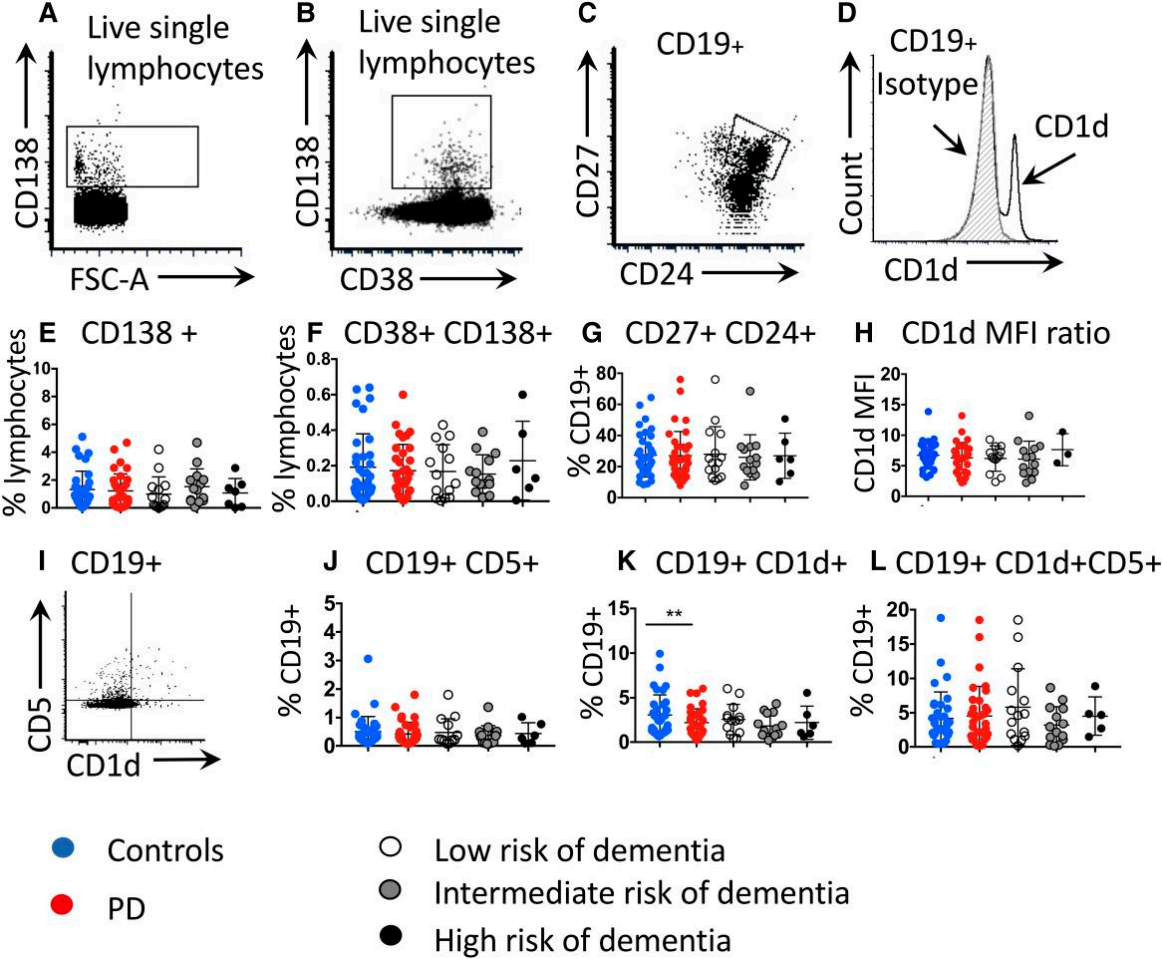
**B lymphocyte subsets in patients with Parkinson’s disease at low and high risk of developing an early dementia.** Plots showing representative gating of plasma cells (**A**) CD138^+^ and (**B**) CD38^+^CD138^+^ cells, (**C**) plasmablast-like cells (CD27^+^, CD24^+^) and (**D**) CD1d staining compared to isotype control. (**E**) Proportions of CD138^+^ plasma cells across groups. (**F**) Proportions of CD38^+^/CD138^+^ plasma cells. (**G**) Proportions of CD27^+^CD24^+^ plasmablast-like cells. (**H**) CD1d MFI ratio (CD1d MFI:isotype MFI). (**I**) Plot showing representative gating for CD5- and CD1d-positive cells. (**J**) CD5^+^ proportions. (**K**) CD1d^+^ cells. **P = 0.017 [paired *t*-test *t*(33) = 2.502]. (**L**) CD1d^+^ and CD5^+^ cells. PD, Parkinson’s disease.

To interrogate whether this reflected a universal reduction across all B-cell subsets or was driven by the loss of a specific subset, we used a variety of canonical B-cell markers to identify transitional (CD27^−^CD24^HI^CD38^HI^) (Fig. 2D and H), intermediate transitional (CD27^−^CD24^int^CD38^int^) (Fig. 2D and G), naïve (CD19^+^CD27^−^, IgD^+^) (Fig. 2E and K), memory (CD27^+^, IgD^−^) (Fig. 2E and I), double negative (CD27^−^IgD^−^) (Fig. 2E and L) and CD27^+^IgD^+^ B cells (Fig. 2J) as well as plasma cells (CD138^+^ and CD38^+^CD138^+^ plasma cells) (Fig. 3A, B, E and F), plasmablast-like cells (CD27^+^CD24^+^) (Fig. 3C and G) and CD5^+^ and CD1d^+^ cells, subsets enriched for regulatory B cells (Fig. 3D, H and I–L). There were no differences between the patient/control groups in the proportions of B-cell subsets apart from a reduction in CD1d^+^ (regulatory) B cells in all Parkinson’s disease patients (not just the high-risk group) compared to matched controls (*P* = 0.017 [paired *t*-test *t*(33) = 2.502] (Fig. 3).

**Figure 4:**
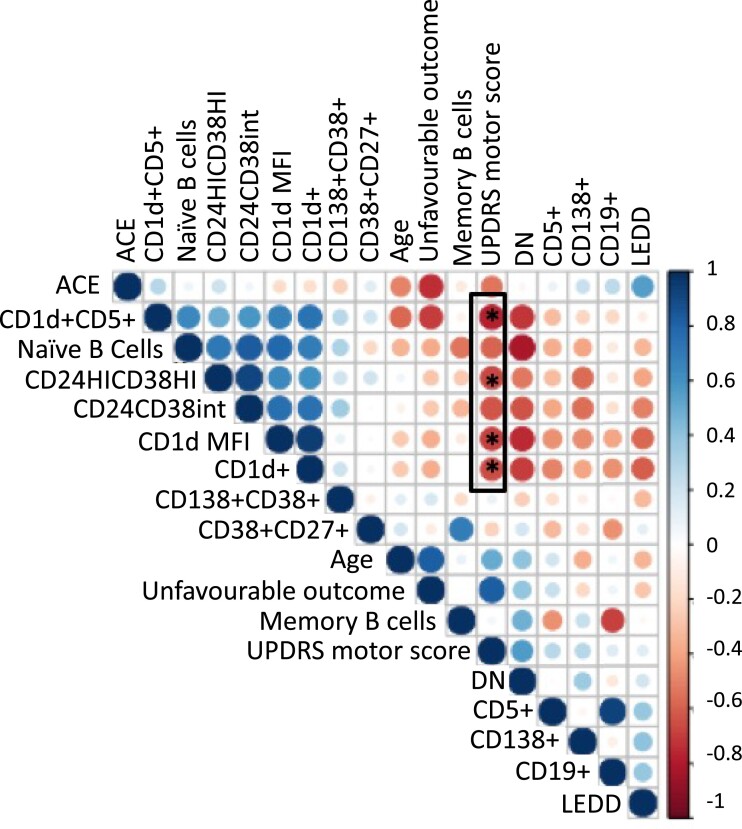
**Heat map showing the correlation matrix of B lymphocyte subsets and clinical variables in patients with Parkinson’s disease.** Memory, memory B cells; DN, double negative (CD27^−^IgD^−^), MDS-UPDRS motor, MDS Unified Parkinson’s Disease Rating Scale part III motor subscore; CD138^+^, plasma cells; CD5^+^, CD5^+^ cells; LEDD, levodopa equivalent daily dose; ACE: ACE-R, Addenbrooke’s Cognitive Examination Revised; Naïve, naïve B cells; CD24^HI^CD38^HI^, transitional B cells (likely IL10-producing population), CD24^HI^CD38^int^ (an additional IL10-producing population); CD1d MFI, CD1d mean fluorescence intensity; PD, Parkinson’s disease. Unfavourable outcome: probability of dementia, postural instability or death at 5 years.^36^ * indicates statistically significant correlations (*P* < 0.05).

Within the patient cohort, several B-cell subsets with regulatory capacity were negatively correlated with the MDS-UPDRS-III scores (Fig. 4) (i.e. higher proportions of these subsets were associated with better motor scores). This included the percentage of CD24^HI^CD38^HI^ transitional B cells (*r* = −0.34, *P* = 0.048), the percentage of CD1d^+^ lymphocytes (*r* = −0.35, *P* = 0.042), the percentage of CD1d^+^CD5^+^ B lymphocytes (*r* = −0.39, *P* = 0.024) and the CD1d MFI ratio (*r* = −0.46, *P* = 0.012) (Supplementary Fig. 2). A regression model was then constructed including all B lymphocyte subsets/markers that were negatively associated with MDS-UPDRS-III scores. The model as a whole explained 37.6% of the variance in MDS-UPDRS-III scores (*R*^2^ = 0.376, SE 10.6) and was a significant predictor of motor severity as evaluated by MDS-UPDRS-III scores [*F*(4,24) = 3.612, *P* = 0.019], although markers within the model did not reach significance at an individual level.

### B cells in Parkinson’s disease patients at higher dementia risk are more responsive to stimulation *in vitro*

**Figure 5:**
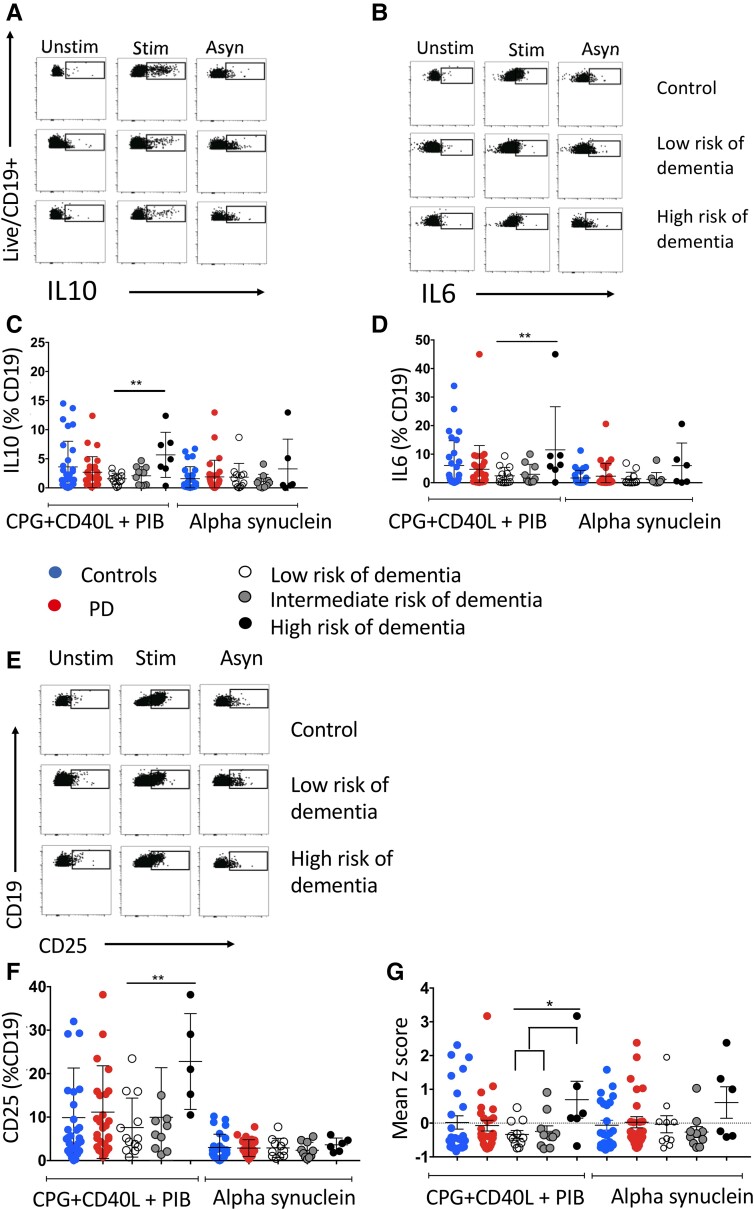
**B lymphocyte stimulation assays showing intracellular staining and activation markers.** (**A**) Post-culture gating of CD19^+^ cells showing IL10 intracellular staining in the unstimulated condition [brefeldin A (BFA) only], stimulated [using PIB, phorbol 12-myristate 13-acetate (PMA) + ionomycin (I) + BFA; CD40L, CD40 ligand; CpG, CpG] and alpha-synuclein conditions. Each point in **A**, **B** and **E** represents a single cell from three separate patients (a control, a patient at low risk of early dementia and a patient at high risk of early dementia). The points in graphs **C**, **D**, **F** and **G** represent each patient or control (referring to the proportion of their cells staining positive for the respective cytokine or intracellular marker). (**B**) Post-culture gating of CD19^+^ cells showing IL6 intracellular staining with unstimulated condition (BFA only), stimulated (as in **A**) and alpha-synuclein conditions. (**C**) Proportion of cells staining positive for intracellular IL10 across risk groups. There was a significant effect of dementia risk group [ANOVA *F*(2,51) = 8.08, *P* = 0.001]; *post hoc t*-tests (Bonferroni) showed this was due to differences between the high-risk group and other groups. (**D**) Proportion of cells with positive intracellular staining for IL6. There was a significant effect of risk group [ANOVA *F*(2,52) = 9.58, *P* = 0.0003]; *post hoc t*-tests (Bonferroni) showed this was due to differences between the high-risk group and other groups. (**E**) Post-culture gating of CD25^+^ cells showing unstimulated, stimulation and alpha-synuclein conditions. (**F**) Proportion of cells staining positive for CD25. There was a significant effect of risk group [ANOVA *F*(2,52) = 7.9, *P* = 0.001] with *post hoc t*-tests (Bonferroni) showing this was due to differences between the high-risk group and other groups. (**G**) Overall stimulation *Z*-scores showing that there was a significant increase in cytokine release in the high-risk group (low versus high risk, Mann–Whitney U = 46, *P* = 0.037; low + intermediate versus high risk, Mann–Whitney U = 26, *P* = 0.03). **P < 0.01.

To profile the pro- and anti-inflammatory cytokine-producing capacity of circulating B cells in Parkinson’s disease patients, we performed *in vitro* stimulation assays, using a standard B-cell stimulation consisting of CpG (a TLR9 agonist), CD40L (mimicking T-cell co-stimulation), ionomycin and PMA or alpha-synuclein fibrils (a disease antigen-specific stimulus) and measured IL10, a regulatory cytokine, or IL6, a pro-inflammatory cytokine (Fig. 5A and B for representative plots). We also measured surface expression of MHC Class II and CD25 (the alpha chain of the IL2 receptor), markers of B-cell activation (Fig. 5E). B cells from patients at high risk of an early dementia produced ‘more’ cytokines than those from the lower risk groups (Fig. 5C and D). CD25 expression post-stimulation was also increased in those at high risk (Fig. 5F). There was no difference between groups in the IL6:IL10 ratio, a parameter previously used to describe the overall balance between pro- and anti-inflammatory B-cell effects^37^ (Supplementary Fig. 3A). MHC Class II expression did not change (see Supplementary Fig. 3B and C).

As IL10, IL6 and CD25 were highly correlated with each other (*r* = 0.8), a composite variable was constructed (‘activation markers’) by converting them into *Z*-scores and using the mean score as an overall measure of activation for each individual. This composite variable was increased in the high-risk group (Fig. 5G). There was no correlation between cytokine production (either individually or the composite score) and overall IgG or B-cell numbers.

### B lymphocytes are reduced in alpha-synuclein transgenic mice compared to controls

**Figure 6:**
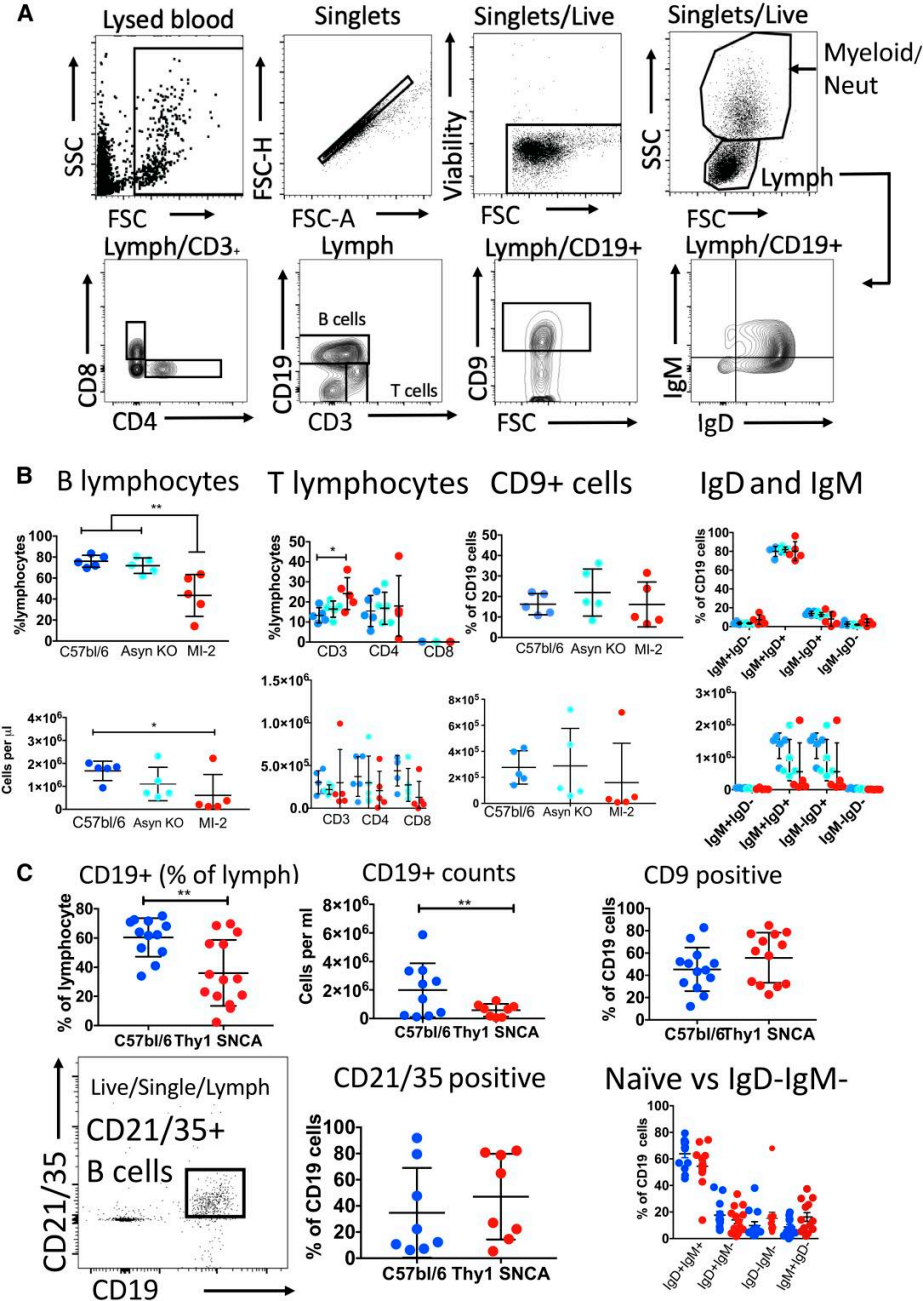
**B- and T-cell numbers in two alpha-synuclein transgenic models of Parkinson’s disease.** (**A**) Representative plots showing gating for PBMCs from mice identifying lymphocytes, CD19^+^ B cells, CD3^+^ T cells (and CD4^+^ and CD8^+^ subsets), CD9^+^ B cells (regulatory) and naïve (IgD^+^IgM^+^/IgD^+^IgM^−^) or memory (IgM^−^IgD^−^) cells. Each point represents a single cell from one mouse. For plots **B** and **C**, each data point represents data from one individual mouse. (**B**) The proportion and absolute counts of B lymphocytes are decreased in the MI-2 mice compared to two different controls (C57/bl6 and Asyn KO). The proportion but not numbers of T cells was increased in MI-2 mice. There were no differences in CD9^+^ and naïve/memory subsets. There were five male mice in each group, all aged between 17 and 18 months. Asyn KO, alpha-synuclein null mice. **P < 0.01 and *P < 0.05. (**C**) The proportion and absolute counts of B lymphocytes are decreased in Thy1 SNCA mice compared to C57bl/6 controls. Proportions c57bl/6 mean 36.6% (SD 22.63) and Thy1 SNCA mean 60.4% (SD 13.15). Absolute counts C57bl/6 mean 1.9 × 10^6^ (SD 1.9 × 10^6^) and Thy1 SNCA mean 0.5 × 10^6^ (SD 0.43 × 10^6^). **P < 0.01. The data were obtained over three separate experiments, from 13 C57bl/6 mice (*N* = 13, 6 females; mean age 19.1 months, SD 1.5 months) and 13 Thy1 SNCA mice (*N* = 13, 8 females; mean age 18.7 months, SD 1.72). There were no differences in the CD9^+^, naïve/memory subsets or CD21/35-positive subsets. Asyn KO, α synuclein knock out.

In order to test whether the observed decrease in B lymphocytes was associated with alpha-synuclein–driven pathology, we measured circulating B lymphocytes in two alpha-synuclein–expressing transgenic mouse strains. The Thy1 SNCA mouse overexpresses human alpha-synuclein under the control of the Thy1 promoter on a C57BL/6 background,^38^ while the MI-2 mouse has a truncated version of human alpha-synuclein that is prone to aggregation and is bred on an alpha-synuclein null background^39^ (Supplementary Fig. 5). We used cell surface markers to identify a limited number of cell subsets (see Fig. 6A). There was a clear decrease in B lymphocytes in both mouse strains compared to the relevant controls (Fig. 6B and C). There were no differences in the cell subsets apart from an increase in the proportion of T cells in the MI-2 mice (with no difference in the absolute cell counts).

### Depleting B lymphocytes either genetically or using a monoclonal antibody to the B cell antigen CD20 results in worse outcomes in the 6-OHDA toxin-based mouse model of Parkinson’s disease

**Figure 7:**
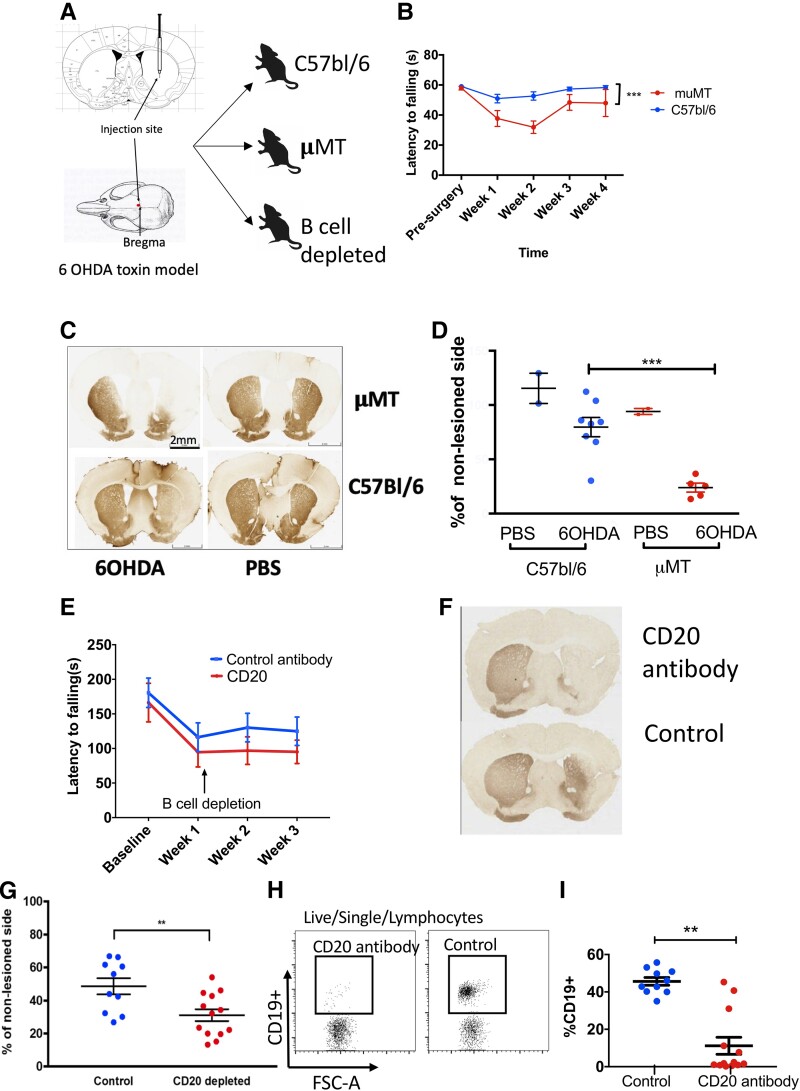
**Pathological and motor outcomes are worse in B-cell–depleted 6-OHDA mouse model of Parkinson’s disease.** (**A**) The 6-OHDA toxin model showing the injection site in the striatum. (**B**) Motor performance on the rotarod test for *N* = 28 μMT mice (15 male) and *N* = 28 C57bl/6 mice (11 male) collected across two experiments (with equal numbers in each group for both experiments). All mice were aged between 11 and 14 weeks at the time of the experiment. The figure shows the mean and standard error of the mean (SEM). Lower latencies suggest poorer performance (mice fall off sooner). This short protocol test has a ceiling effect (a limit of 60 s on the bar) meaning that the C57bl/6 did not show a significant change after surgery. A two-way ANOVA was undertaken with genotype and week of testing as independent variables and showed that there was a significant main effect of genotype overall [*F*(1,133) = 26.65, *P* < 0.0001] and of time [*F*(4,133) = 10.33, *P* < 0.0001] with a significant interaction between the two terms [*F*(4,133) = 3.02, *P* = 0.02]. (**C**) Representative histological image showing TH^+^ staining with a more extensive loss of dopaminergic fibres in the μMT mouse (examples from two individual mice: one μMT and one C57bl/6 mouse; both female, 12 weeks). (**D**) Quantification of the mean optical density of staining in the striatum (taken from 6–10 sections per mouse) showing the staining as a percentage of the non-lesioned side (*N* = 8 C57bl/6, *N* = 5 μMT) from mice culled 4 weeks after surgery. Each point represents one individual. Unpaired *t*-test, *t*(11) = 4.07, *P* = 0.0006. (**E**) Motor performance on the rotarod test for mice given CD20 antibody (*N* = 10) and control antibody (*N* = 13) female mice aged between 22 and 26 weeks. A two-way ANOVA with treatment and week of testing as independent variables showed there was a significant main effect of week of testing [*F*(3,72) = 9.44, *P* = 0.0001]. There was no main effect of treatment type [*F*(1,24) = 1.05, *P* = 0.32]. (**F**) Representative histological image showing TH^+^ staining with more extensive loss of dopaminergic fibres in a mouse given a CD20 antibody versus a mouse given the control antibody (examples from two individual mice). (**G**) Quantification of the mean optical density of staining (across 6–10 sections for each mouse) in female C57/bl6 mice (12 weeks old) given either control (*N* = 10) or an anti-CD20 monoclonal antibody (*N* = 13). Unpaired *t*-test, *P* = 0.01. (**H**) Representative flow cytometry gating plot showing B cell (CD19^+^) gating in a mouse given the control antibody and a mouse given the CD20 antibody (peripheral blood). Each point represents a single cell. (**I**) B cell percentages in individual mice calculated from flow cytometry plots in the CD20 and control groups. Unpaired *t*-test, *t*(21) = 6.22, *P* = 0.001. TH, tyrosine hydroxylase.

We used a toxin-based model of dopaminergic cell death that recapitulates the core neuronal loss and microglial activation seen in patients with Parkinson’s disease using an injection of 6-OHDA directly into the striatum (Fig. 7A). Mice were injected in the right striatum, and the contralateral side of the brain was used as an internal control for cell quantification/staining. We compared motor and histological outcomes in μMT mice (that are deficient in mature B lymphocytes) and in mice given a CD20 monoclonal antibody (that depletes mature B cells) 1 week after surgery to investigate the effect of decreased B lymphocytes on disease course. Mice were culled 4 weeks after surgery. C57BL/6 (wild-type) mice were used as controls in both experiments. The μMT mice had significantly worse motor outcomes than controls (Fig. 7B) and more extensive dopamine loss (see Fig. 7C and D). As the T-cell compartment is also affected in this model (due to abnormal development of lymphoid tissue in the absence of B cells^40^), we also depleted B cells in wild-type mice. C57BL/6 mice were given a CD20 monoclonal antibody 1 week following surgical intervention had no statistically significant difference in motor phenotype compared to controls treated with a human anti-CD20 antibody (Fig. 7E) but did have more extensive dopamine loss (see Fig. 7F and G). Peripheral B-cell depletion is confirmed in Fig. 7H and I. Intensity of Iba1 staining (a marker of microglial activation) and quantification of microglia (as a percentage of the non-lesioned side) were not different between μMT and control mice (see Supplementary Fig. 4A and B).

## Discussion

In summary, we have found that changes in the B-cell compartment are associated with disease development and progression. There are higher levels of alpha-synuclein antibodies in RBD cases at high risk of Parkinson’s disease conversion and B cells are lower in number and more reactive to stimulation in Parkinson’s disease patients at risk of an early dementia. In alpha-synuclein mouse models, we showed that circulating B cells were also decreased suggested that this a result of alpha-synuclein pathology. We also found that regulatory subsets may play a protective role potentially by attenuating the pro-inflammatory responses contributing to disease progression. Greater proportions of these subsets were associated with better motor outcomes. Knocking out B cells (either genetically or with an anti-CD20 antibody) in a toxin-based model resulted in worse outcomes suggesting that the protective effect (potentially of regulatory cells) is prominent early in disease.

We have shown for the first time that serum antibodies to a pathological form of alpha-synuclein (fibrils) are elevated in individuals with RBD who are at high risk of progression to overt Parkinson’s disease. This early humoral immune response to pathological alpha-synuclein evident in the prodromal phase of Parkinson’s disease is consistent with a recent study describing more prominent alpha-synuclein–specific T-cell responses in Parkinson’s disease patients prior to disease onset and shortly after diagnosis, compared with those later in the disease course.^8^ Interestingly, we did not find a difference in alpha-synuclein antibody titres between the Parkinson’s disease group and controls despite previous studies suggesting that alpha-synuclein antibodies are elevated in early disease (<5 years), albeit inconsistently (reviewed in Scott *et al.*^7^). We made every effort to reduce clinical heterogeneity in this Parkinson’s cohort, which could have contributed to our negative findings, although there was still some variability in disease duration within our cohort (mean 3.2, SD 2.6). Alternatively, it may be that a more sensitive analysis of immunoglobulin subtypes is required to show a more nuanced antibody signature (as suggested in Folke *et al*.,^41^ who describe a complex phenotype in multiple system atrophy versus Parkinson’s disease versus controls). Importantly, our study did find a difference compared to healthy controls, but at a much earlier time point in the disease, namely in the prodromal phase, consistent with the overall hypothesis that antibody responses are increased in early disease but decrease as the disease progresses.

Therapeutic trials of alpha-synuclein antibodies in Parkinson’s disease are underway^42^ with initial data showing a decrease in blood and CSF alpha-synuclein levels. Antibodies may have beneficial effects, mediating the clearance of some pathological forms of alpha-synuclein (for example via Fcγ receptor-mediated phagocytosis). Alternatively, antibodies could contribute to disease progression by promoting an inflammatory milieu in the brain (e.g. via activation of the complement cascade), leading to microglial activation, peripheral immune cell recruitment and further neuronal death.

We also investigated peripheral blood B-cell number and phenotype in patients with established Parkinson’s disease. In line with previous studies, we found a decrease in total B-cell number compared with controls,^22,23,25^ but here we describe (for the first time) that this observation is driven by a marked decrease in B cells in the patient subset at highest risk of developing an early dementia. The B cells remaining in these high-risk patients also showed a greater cytokine response (both IL10 and IL6) when stimulated *ex vivo* compared with controls consistent with recent studies^26^ and also had higher expression of the activation marker CD25, consistent with *in vivo* priming. Thus, in this subgroup of patients, the B cells are both depleted in number but more activated, suggesting that they may play a role in driving pathology in those with a worse prognosis. The decrease in circulating B lymphocyte numbers was not clearly driven by a decrease in any one subset (rather appearing to be a global reduction in B cells). This finding was recapitulated in two alpha-synuclein transgenic mouse models of Parkinson’s disease, suggesting that this is associated with a response to pathological alpha-synuclein rather than other parameters relating to disease in humans.

All of this data would therefore suggest that that B cells are activated in Parkinson’s disease, particularly in those at highest risk of disease progression and that this is associated with a reduction in circulating B cells, either due to their exit from the blood (potentially to the CNS or gut, where alpha-synuclein pathology is well-described^43^) or due to activation-induced cell death. In one recent study, Yan *et al*.^26^ found a decrease in CXCR3^+^ B cells that may reflect homing to lymphoid tissue or the menginges by these cells. Further work is required to delineate between these possible explanations.

Our analysis of B-cell subsets also generated some interesting observations: Parkinson’s disease patients in our cohort had fewer CD1d positive cells than their matched controls. We also noted that a higher proportion of circulating CD24^HI^CD38^HI^ transitional B cell and other subsets enriched for ‘regulatory’ IL10-producing B cells, including CD1d and CD5^+^ cells,^16^ was associated with better motor scores (lower MDS-UPDRS-III scores). Interestingly, in mice, CD1d-mediated presentation of lipid antigens by regulatory B cells to invariant NKT cells resulted in suppressive iNKT capable of modulating pathological autoreactive Th17 and Th1 CD4 T cells.^44^ Our data therefore suggest that B cells with regulatory capacity may be beneficial in Parkinson’s disease, although whether that requires CD1d or iNKT activation by B cells or is due to direct effects on pathological T cell responses via IL10 production will need further study. Regardless of the mechanism, our observations are consistent with the previously described protective effects of T regulatory cells in Parkinson’s disease (reviewed in Chen *et al*.^45^) and the more recently described decrease in functional regulatory B cells (as characterized by surface markers and *in vitro* effector function) in Parkinson’s disease compared to controls.^27^ This suggests that boosting regulatory T or B lymphocytes may be a useful therapeutic strategy to prevent disease progression. We would argue that it is the balance between the effector and regulatory subsets that results in a protective or deleterious effect.

In this respect, the study we undertook using a toxin-based mouse model supported the suggestion from our human immunophenotyping data that some B-cell subsets may play a protective role in Parkinson’s disease. We found that B-cell deficiency led to worse behavioural outcomes. B-cell deficiency or antibody-based depletion was associated with a larger dopaminergic cell loss. Previous studies have examined the effect of lymphocyte deficiency on motor and histological phenotypes in toxin-based models of Parkinson’s disease, one using athymic rats^46^ and another using RAG^−/−^ mice that lack both T and B lymphocytes.^47^ Both of these studies noted worse outcomes in lymphocyte-deficient mice rescued by bone marrow transplantation.^47^ Depleting CD4^+^ T cells alone appears to improve outcomes in animal models.^48^ Taken together with our results, one could hypothesize that the rescue obtained with bone marrow transplantation in lymphocyte-deficient mice is at least in part mediated by the protective effects of regulatory B cells. Further work examining the effect of transferring B lymphocytes enriched for a regulatory phenotype is required. We also acknowledge that an isotype control would be a more appropriate control for the antibody depletion than the chimeric version of the CD20 antibody (rituximab). Human IgG Fc from the constant portion of the antibody has excellent binding to mouse Fcγ receptors therefore is still a useful control for non-specific Fc-dependent antibody effects. The overall protective effect of B cells at least in this model shows that they have an action that is independent of alpha-synuclein pathology and related in some way to cell death and/or microglial activation.

The strengths of this study include the provision of well-matched age, gender and genotype controls for both a prodromal RBD and early Parkinson’s disease patient cohort stratified by risk of disease progression as well as the intensity and depth of analysis we have undertaken looking at different types of B cells and their function. Previous studies have been limited by the heterogeneity of clinical cohorts and sub-optimal control groups.^7^ We have also focussed on identifying immunological factors associated with clinical progression rather than diagnosis, namely RBD to Parkinson’s disease conversion and risk of progression to an early dementia in established Parkinson’s disease, utilizing our well-validated markers to identify patients at risk of worse outcomes and showing that changes in the B-cell compartment are related to risk of disease progression. Potential confounding factors in our study include the effects of dopaminergic medication on immune cells, as most immune cells express dopamine receptors.^49^ The study is also limited by the relatively small sample number (although similar to published literature) and the lack of longitudinal follow up. In future work, we would like to explore B-cell phenotypes in prodromal Parkinson’s disease cases, to allow us to determine how early in the disease the observed functional B-cell changes occur, and to explore the relationship between antibodies and B-cell phenotypes in more detail. However, in our current study, we only had access to stored serum samples in the REM sleep behaviour cohort.

Overall, our results highlight that changes in the B-cell compartment are associated with disease progression and raise the possibility that therapeutic strategies augmenting regulatory B cells may have utility in very early Parkinson’s disease or RBD.

## Supplementary Material

fcad060_Supplementary_Data

## Data Availability

Data are available on request.

## Abbreviations

ACE-R =: Addenbrooke’s Cognitive Examination Revised
BAFF =: B-cell activating factor of the tumour necrosis factor receptor family
BSA =: bovine serum albumin
CRP =: C-reactive protein
CV =: coefficient of variation
GM-CSF =: granulocyte macrophage colony-stimulating factor
IL6 =: interleukin 6
IL10 =: interleukin 10
MAPT =: microtubule-associated protein tau
MDS-UPDRS =: Movement Disorder Society Unified Parkinson’s Disease Rating Scale
MHC =: major histocompatibility complex
MMSE =: Mini-Mental State Examination
MSD =: Mesoscale Discovery
NIHR =: National Institutes of Health Research
6-OHDA =: 6-hydroxydopamine
PAMP =: pathogen-associated molecular motif
PBMC =: peripheral blood mononuclear cell
PBS =: phosphate-buffered saline
PFA =: paraformaldehyde
PMA =: phorbol 12-myristate 13-acetate
RBD =: REM sleep behaviour disorder
REM =: rapid eye movement
RPM =: revolutions per minute
SNCA =: alpha-synuclein
TH =: tyrosine hydroxylase
UPDRS =: Unified Parkinson’s Disease Rating Scale
