# Supplementary material for: B lymphocyte responses in Parkinson’s disease and their possible significance in disease progression
Source: Brain Commun. 2023 Mar 9;5(2):fcad060. doi: 10.1093/braincomms/fcad060 (PMC10042276; doi:10.1093/braincomms/fcad060)

Supplementary materials and methods

PD patient risk stratification

The stratification was done on the basis of their MAPT (tau) genotype, pentagon copying ability and semantic fluency score (the high risk group was defined as MAPT H1/H1 genotype and any one of either semantic fluency <20 in 90s or pentagon copying score <2, the intermediate group had any one of these factors and the low risk group had none)(as in Williams-Gray et al.[1] which used the same patient cohort). Their overall probability of a 'poor outcome' (either dementia, postural instability or death) was also calculated using a combination of age, UPDRS axial score and semantic fluency score.[2]

PD patient clinical assessments

All patients went through a full clinical assessment including medical history and co-morbidities, the Movement Disorder Society Unified Parkinson's Disease Rating Scale (MDS-UPDRS), the Addenbrooke's Cognitive Examination (ACE-R), assessment of semantic fluency (animal naming in 90s), pentagon copying and the Beck Depression Inventory (BDI).

Antigen preparation for antibody study

Alpha synuclein monomers (from rPeptide, S-1001-2) were suspended in MilliQ water. Using the Amicon Ultra 3K, samples were concentrated and resuspended to make a solution of 10mM Tris-HCl, pH 7.6, 5µg/µl concentration. The tubes were then placed in a 37°C thermomixer and shaken for 7 days at 1,000 RPM. Fibrils were stored in aliquots of 20µl at -20°C. Prior to use, they were defrosted and sonicated with 60 pulses and 10% power to ensure that the aggregates were not too large.

Alpha synuclein fibrils were produced using the method described above (using alpha synuclein from rPeptide S-1001-2). The phosphorylated S129D peptide was obtained from Abcam (ab188826). The fibrils and S129D peptide were biotinylated using the EZ link Sulfo NHS biotin kit as per the manufacturer's instructions (21335). The proteins were dialysed after biotinylation to remove excess biotin using Slide-A-Lyzer dialysis cassettes (66205) and

associated float buoys (Thermofisher 66430) as per manufacturer's instructions. Biotinylation status was confirmed using the Pierce biotin quantitation kit (28005). The tau protein and Y39 were obtained from Creative Biomart as custom biotinylated protein/peptide (tau: MAPT-38H lot 290831, Y39: GKTKEGVLYVGSKTK lot 100281). Recombinant alpha synuclein was available in a biotinylated form commercially (alpha synuclein with biotin Anaspec AS-55581). The manufacturers of all the proteins confirm that the products are purified to remove endotoxin. The antigens were coated on the plates at a concentration of 0.15 picomole per well with the exception of tau which was coated at a concentration of 0.3 picomole per well. The antigens were diluted in 1% BSA (Probumin. Millipore, cat. no. 82-045-1).

Seven point standard curves were constructed using known duplicate concentrations of commercial mouse or rabbit antibody to the protein or peptide in question. All plates included 2 blank wells. For the alpha synuclein fibrils and recombinant alpha synuclein, mouse anti-alpha synuclein (Abcam, ab1903) was used at a starting concentration of 2000ng/ml with serial 1 in 5 dilutions. For the tau protein, mouse anti-tau antibody (Abcam, ab80579) was used at a starting concentration of 2000ng/ml with serial 1 in 5 dilutions. For the S129D peptide, a rabbit anti S129D antibody was used (Abcam, ab51253) at a starting concentration of 2840 ng/ml with serial 1 in 7 dilutions. There was no commercial antibody to Y39 and therefore it was not possible to generate a standard curve for this antigen. Instead, raw signal intensity scores were normalised to a standard serum sample which was the same across assays. The concentrations of antibodies for the standard curves were optimised prior to running the test assays on patient samples.

Blood sampling in PD patients

Up to 50mL of venous blood was collected from each patient or control using a combination of lithium heparin tubes for peripheral blood mononuclear cell (PBMC) isolation, clotted tubes for serum collection and ethylenediaminetetraacetic acid (EDTA) tubes for full blood counts. Clotted samples were left at room temperature for 15 minutes before centrifuging at 2000RPM for 15 minutes.

Serum processing

Venous blood samples for all subject groups were collected in plain serum tubes and allowed to clot for 10 to 15 minutes prior to centrifugation. PD and control samples were centrifuged

at 2000 rpm for 15 minutes; blood samples from the RBD group were centrifuged at 3700 rpm for 10 minutes. Serum was removed and stored in aliquots at -80°C until assays were performed. Serum samples were thawed on wet ice on the morning of experiments.

Ficoll gradient separation and freezing of cells

The PBMC layer was removed and washed twice with PBS. Depending on the total number of cells, excess cells were frozen in freezing medium (90% foetal calf serum [FCS, Sigma Aldrich F7524] and 10% dimethyl sulfoxide [DMSO, Sigma Aldrich D8418]) and placed in a cryopreservation chamber at -80°C.

Cells were resuspended at a concentration of 1 x 10^6 per 100µl of FACS buffer for staining plus 2% mouse serum (0.1% BSA, Probumin. Millipore, cat. no. 82-045-1, 0.01% sodium azide, Sigma Aldrich, cat. no. S2002 made up in Phosphate Buffered Saline [PBS], mouse serum Sigma Aldrich, cat. no. M5905).

Cell surface antibodies used for peripheral B cell phenotyping study

The following antibodies markers to cell surface antigens were added: BV786 CD19 (3:100) (BD bioscience 563325), FITC CD5 (2:100) (BD bioscience 555412), BV605 CD24 (5:100) (BD bioscience 562788), BUV395 CD27 (3:100) (BD bioscience 563816), APC CD38 (10:100) (BD bioscience 555462), PE IgM (10:100) (BD bioscience 555783), V450 CD138 (5:100) (BD bioscience 563098),APC H7 IgD (2:100) (BD bioscience 561305), PerCp Cy5.5 CD1d (2:100) (Biolegend 350312), live/dead zombie aqua (0.5:100).

Cell defrosting protocol

PBMCs were removed from the freezer and placed on wet ice with 1mL of 10% FCS until thawed enough to be transferred to a 15ml Falcon tube containing 10% FCS. The tubes were centrifuged at 400g for 10 minutes and then resuspended in 5mL of 10% FCS for cell counts. The tubes were then spun again at 350g for 5 minutes and resuspended in RPMI cell culture

media with glutamine (Gibco 21875-034) with 10% heat inactivated foetal calf serum to a concentration of 0.25 x 106 cells per 200µl.

Antibodies used for B cell stimulation study

The following antibodies were added BV786 CD19 (3:100) (BD bioscience 563325), FITC IL10 (5:100) (, BUV395 CD25 (2:100), APC CD38 (10:100), PE IL10 (5:100) (BD bioscience 559330), AF700 MHC Class II (2:100) (BD biosciences 560743), V450 CD138 (5:100) (BD biosciences 562098), viability stain (0.5:100) (Biolegend 423101).

Antibodies used for mouse blood staining

The following antibodies were used to stain peripheral blood PBMC from mice: FITC CD8 Biolegend 100706, PerCPefluor IgM eBioscience 46-5790-82, PE CD21/35 biolegend 123410, PE dazzle IgD 405742, PECy7 CD4 biolegend 100528, efluor450 CD9 eBioscience 48-0091-82, BV785 CD19 biolegend 302240, live dead zombie UV biolegend 423107).

Mice

Blood sampling

Blood samples were obtained from the tail vein (Thy1 SNCA mice and controls) or from post mortem cardiac puncture (MI-2 mice and controls) into heparin coated tubes. Red cell lysis was performed on blood samples using red cell lysis buffer for 1 minute (155mM NH4CL, 12mM NaHCO3 and 0.1mm EDTA). Subsequent processing was done on ice.

Mouse welfare: 6OHDA surgery

Following surgery, the needle was withdrawn over 2-3 minutes and the animals were allowed to recover. See supplementary methods for additional details regarding recovery from surgery.Once recovered post surgery, mice were returned to their home cages and monitored closely for the next seven days, including daily weights. They were given mash and Nutella for four days post-operatively to encourage them to eat as this model is associated with weight loss due to the effects on the lateral hypothalamus (and the drive to eat). They were given 1ml of sub-cutaneous fluid twice daily for forty eight hours following surgery.

Rotarod testing protocol

Training was done on day one at two different speeds (16rpm and 24rpm) for a total of four trials. If a mouse fell off, it was put on again up to a maximum of one minute. Mice were given a break of at least twenty minutes between trials. Latency to falling was recording in seconds as well as the speed of rotations (the mouse holds on to the rod rather than walking on it). During training all mice were able to stay on the rotating rod for at least a minute on at least one occasion. Training was done over 3 days. Testing was then completed at 30rpm with scores recorded across three trials. The test days were done a week apart for 4 weeks post surgery.

Mouse culling

Mice were culled using a lethal overdose of intraperitoneal pentobarbitone sodium (Merial, UK, 200mg/mol) followed by intracardiac perfusion with ice cold phosphate buffered saline (PBS). After PBS perfusion, the animals were perfused intracardially with 4% paraformaldehyde (PFA) (made from paraformaldehyde powder Sigma Aldrich 158127). The brains were removed from the skull and placed in 4% paraformaldehyde (PFA) overnight for histology. They were then placed in 30% sucrose for 48 hours.

Brain sectioning

The fixed brain was serially sectioned at 30µl with every 6th section stored in the same well in a 24 well plate using a sledge microtome (Leica SM1400). The sections were stored in anti-freeze solution at 4°C for subsequent staining (0.02M Na_2HPO_4, 0.01M NaH_2PO_4,, 0.3% ethylene glycol [Sigma E9129], 0.3% glycerol [Sigma G7893]).

Immunohistochemistry of brain sections

Sections were quenched with 3% hydrogen peroxide/10% methanol for 10 minutes. They were then washed three times before being blocked with 5% rabbit serum in 0.2% triton for 1 hour at room temperature. Sheep anti-mouse tyrosine hydroxylase was added (Millipore 1542) at a concentration of 1:200 over night or rabbit anti-Iba1 (Wako SAF5299, 1:500 overnight). Sections were then washed and biotinylated rabbit anti-sheep antibody (1:1000) or sheep anti-rabbit antibody (1:500) in 5% horse serum was added and incubated at room temperature for an hour. Secondary antibody was washed off and the sections were then incubated with ABC avidin/biotin reagent (Vectastain ABC Kit, Vector Laboratories) for 45 minutes. Sections were

then washed again. Diaminobenzidine solution (Sigma D4293) tablets were added until the staining was clear at which point the sections were washed. They were then dehydrated using 50%, 75%, 90% ethanol and xylene before being mounted in DPX mounting medium (Fluka).

Stereological estimation of TH+ density

Sections from each mouse were mounted in anatomical order (caudal-rostral). Each animal had 5-6 sections from the substantia nigra (SN). Estimations of the number of cells in the SN were performed using a standard methodology (Olympus CAST grid system).[3,4] Briefly, a vertical line was drawn through the most medial tip of the cerebral peduncle that then formed the medial border (excluding the ventral tegmental area). The dorsal border included all of the TH+ cells with the ventral border following the cerebral peduncle until it met the medial vertical line. Between 5 and 6 sections of SN were counted per brain. A 4x objective was used to define the region of interest which was then visualised under a 40x objective. The number of TH+ cells in each counting frame (90 µm x 90 µm) was recorded. Estimated section thickness after processing (dehydration, mounting) was 20 µm. Depth (z section) data was used to create a frequency-distribution curve. A final estimation was performed using the optical fractionator formula to allow for variable antibody penetration.[4] Optical density of TH+ (and Iba1+) staining was done using 6-10 coronal slices at the level of the striatum. Non-specific background was calculated by measuring the optical density at the corpus callosum and finally normalising to the white background.

Quantification of microglial Iba1+ staining

The optical density of Iba1+ staining was done using 6-10 coronal slices at the level of the striatum. Non-specific background was calculated by measuring the staining at the corpus callosum and finally normalising to the white background. DAB-stained slides imaged using the Brightfield Scanner were used to calculate number of microglia. To cover most of the striatum, 10 regions with an area of 500x500µm2 was selected for both the lesioned and non-lesioned side of each midbrain, such that 20 sites were visited for each striatal section. The Cell counter plugin on Qupath was used to point-count the Iba1+ microglia per specified area and subsequently, the mean number of Iba1+ microglia on either the lesioned or non-lesioned side was calculated across data from 3 striatal sections per mouse.

Supplementary Results

Supplementary Figure 1: A) Correlation matrix of variables and markers measured in the prodromal RBD cohort. B) Correlation matrix of variables markers and clinical outcomes in the PD cohort. The heatmap represents values of Pearson's R. * $p<0.05$ ** $p<0.001$. Abbreviations: (abs) = antibodies, Alpha syn = alpha synuclein, MDS probability = Movement Disorders Society score probability of conversion to PD, MDS-UPDRS III = Movement Disorders Society Unified Parkinson's disease rating scale motor subscale, BAFF = B cell activating factor, UPDRS III = Unified Parkinson's Disease Rating Scale III (motor sub scale), BDI = Beck Depression Inventory, MMSE = mini-mental state examination, ACE = Addenbrooke's cognitive examination, LEDD = Levodopa equivalent daily dose, CRP = C-Reactive protein.

Supplementary Figure 2: Scatter plots showing correlations between transitional, CD5+CD1d+, CD1d+ and CD1d MFI and MDS-UPDRS part III motor score in the PD cohort.

Supplementary Figure 3: Additional data for B cell stimulation experiments
A) IL6:IL10 ratio post stimulation across groups showing no significant differences. One way ANOVA $F[3,55]1.41$, $p = 0.025$. B) Representative histograms showing MHC Class II staining across groups following stimulation in 3 individuals (representative plots from control, low risk, intermediate and high risk of dementia). C) There were no significant differences in MHC Class II MFI across groups. Each point refers to the MHC class II MFI for each patient. One way ANOVA $F[3,55] = 7.9$, $p = 0.51$. 'Low risk' and 'High risk' in B refer to dementia risk.

Supplementary Figure 4: Microglia in the 6OHDA model
A) Optical density of microglial staining as a percentage of the non-lesioned side (Unpaired t test, $t [15] = 1.04$, $p = 0.31$). B) Microglial counts as defined by the number of cells staining with Iba1 showing the expected differences between the lesioned and non-lesioned sides but no differences between controls and CD20 depleted animals. Two way ANOVA (with treatment and lesion v no lesion as independent variables). No main effects and no interactions.

Supplementary Figure 5: Transgenic mouse models
Figure shows transgenic models used. The Thy1 SNCA mouse overexpresses human alpha synuclein under the control of the Thy1 promoter on a C57BL/6 background,[5] while the MI-2 mouse has a truncated version of human alpha synuclein that is prone to aggregation and is bred on an alpha synuclein null background.[6]

References

1. Williams-Gray, C. H. *et al.* Abnormalities of age-related T cell senescence in Parkinson's disease. *Journal of Neuroinflammation* **15**, 1–8 (2018).
2. Velseboer, D. C. *et al.* Development and external validation of a prognostic model in newly diagnosed Parkinson disease. *Neurology* **86**, 986–93 (2016).
3. Guillery, R. W. & Herrup, K. Quantification without pontification: choosing a method for counting objects in sectioned tissues. *The Journal of comparative neurology* **386**, 2–7 (1997).
4. Stott, S. R. W. & Barker, R. a. Time course of dopamine neuron loss and glial response in the 6-OHDA striatal mouse model of Parkinson's disease. *The European journal of neuroscience* **39**, 1042–56 (2014).
5. Chesselet, M.-F. *et al.* A progressive mouse model of Parkinson's disease: the Thy1-aSyn ('Line 61') mice. *Neurotherapeutics : the journal of the American Society for Experimental NeuroTherapeutics* **9**, 297–314 (2012).
6. Wegrzynowicz, M. *et al.* Depopulation of dense α-synuclein aggregates is associated with rescue of dopamine neuron dysfunction and death in a new Parkinson's disease model. *Acta Neuropathol* **138**, 575–595 (2019).

Supplementary Table 1: Number of samples remaining after removal of samples with CV exceeding 30% in each assay (number of samples removed)

Assay	Control	Low-risk RBD	High-risk RBD	PD
α-synuclein	49(1)	27(3)	46(3)	50(0)
α-synuclein fibrils	48(2)	30(0)	43(6)	48(2)
S129D	38(12)	24(6)	42(7)	43(7)
Y39	47(3)	30(0)	48(1)	49(1)
Tau	47(3)	24(6)	32(17)	49(1)
IgG	42(8)	27(3)	41(8)	46(4)
CRP	49(1)	30(0)	48(1)	47(3)
BAFF	40(10)	30(0)	46(3)	44(6)

Supplementary Table 2: Mean intra-assay and inter-assay CV for all assays performed

Assay	Mean assay CV (%)	Inter-assay CV (%)
α-synuclein	6.27	71.69
α-synuclein fibrils	6.72	136.89
S129D	7.09	67.07
Y39	4.65	5.07
Tau	8.77	62.37
IgG	8.14	61.85
CRP	6.67	56.20
BAFF	10.34	36.56

Supplementary Figure 1

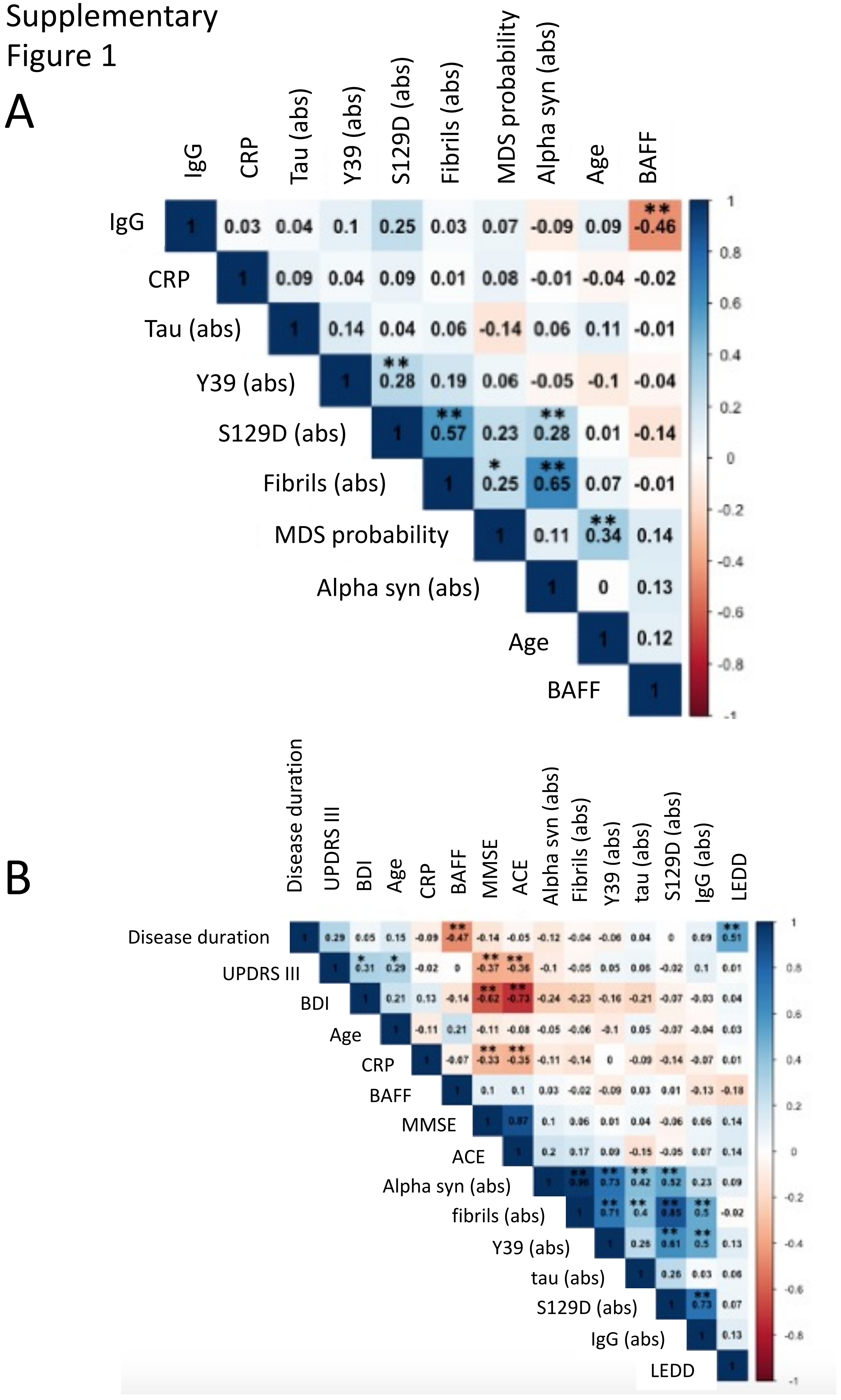

Supplementary
Figure 2

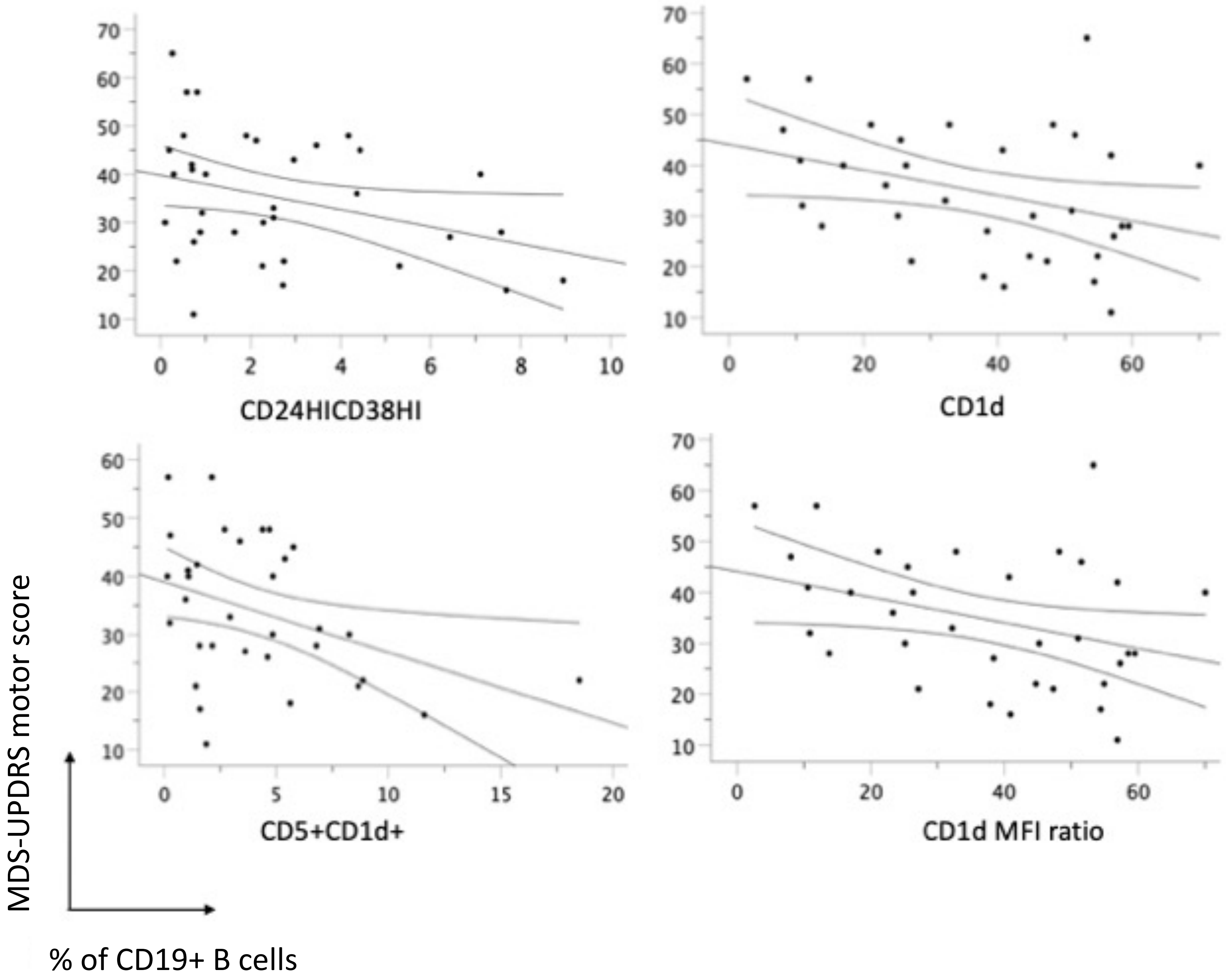

Supplementary Figure 3

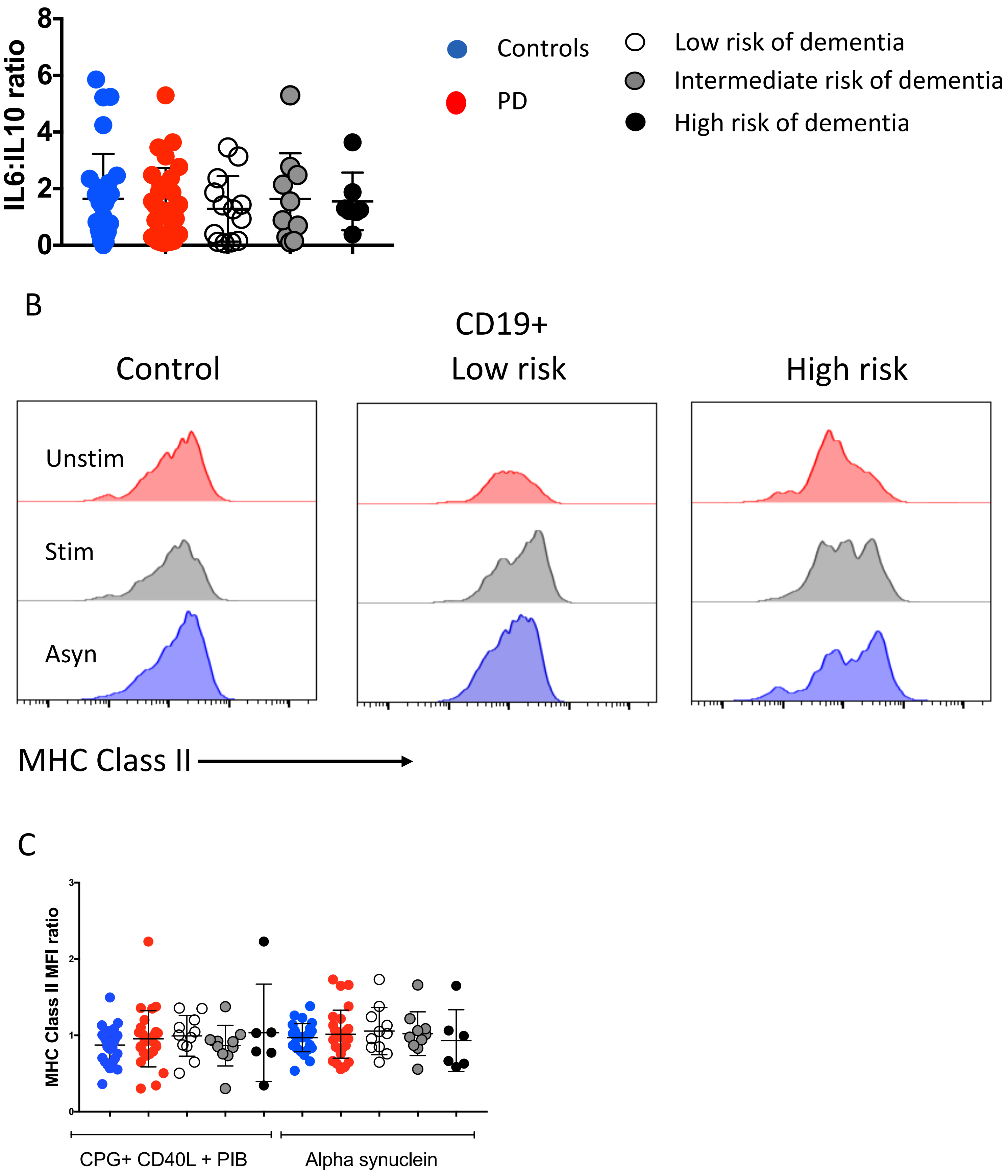

Supplementary
Figure 4

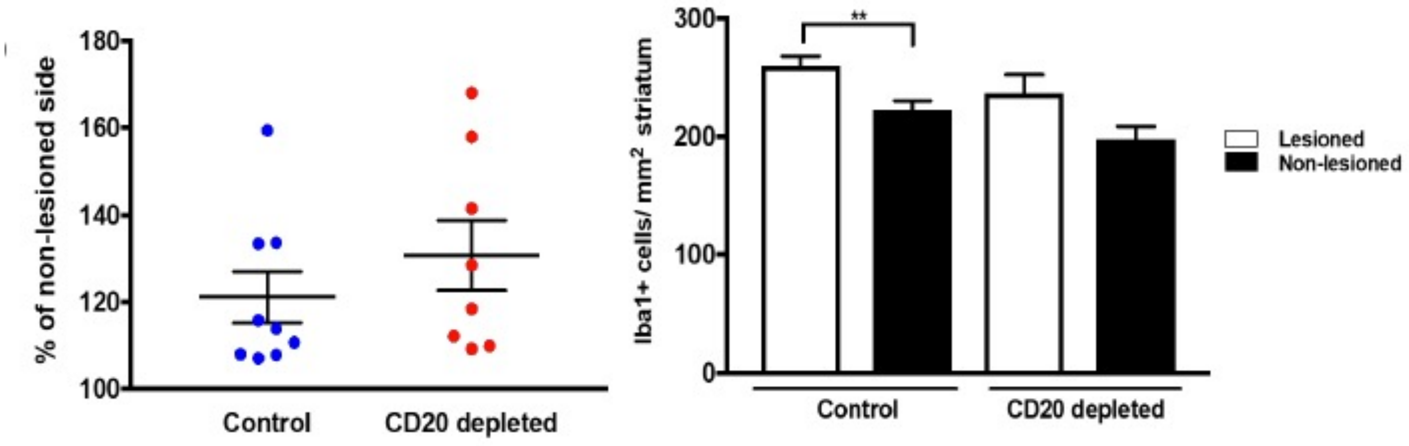

A

B

Supplementary
Figure 5

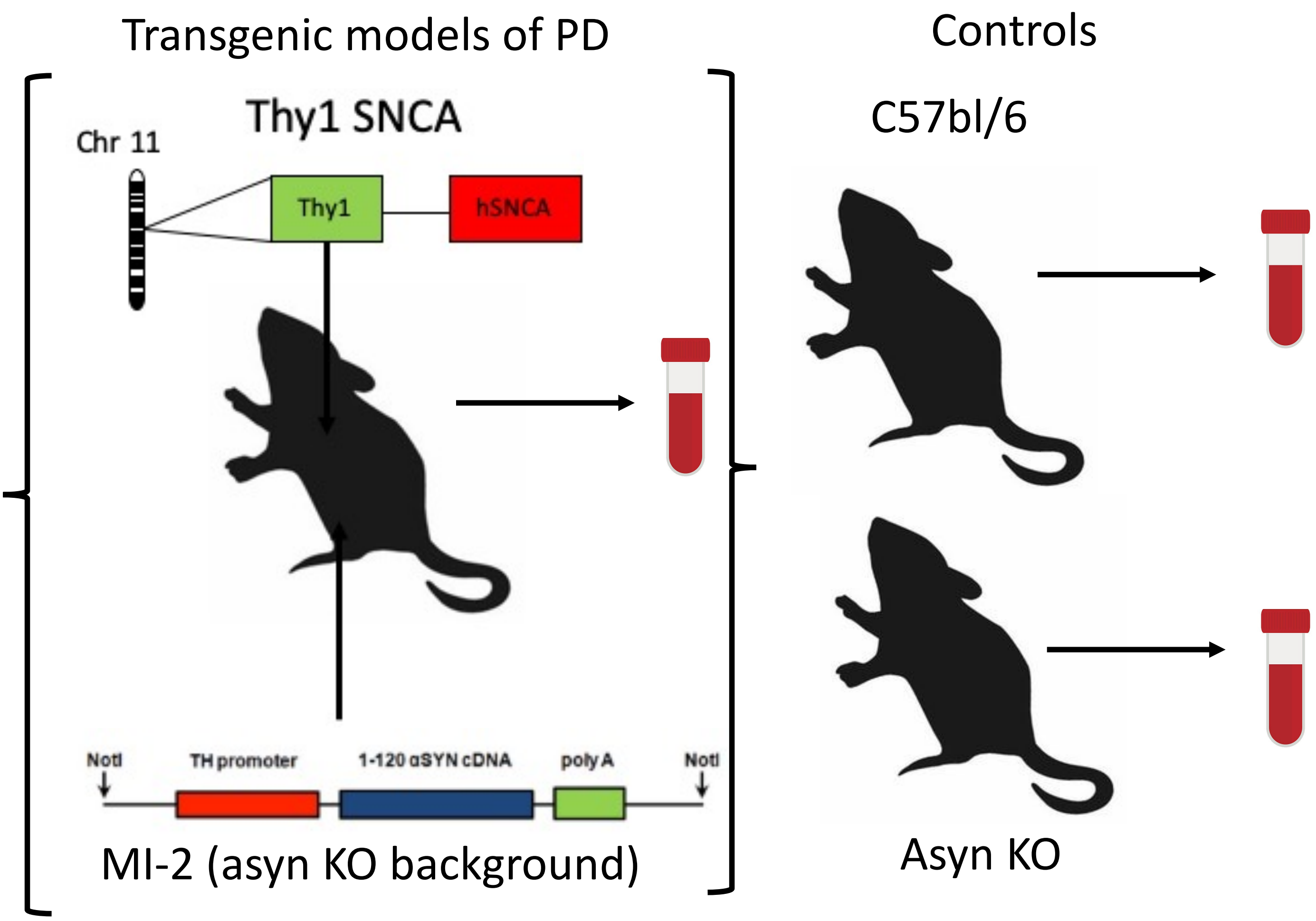

Supplement: fcad060_Supplementary_Data [file fcad060_supplementary_data.pdf]
